# Disparities in postoperative opioid prescribing by race and ethnicity: an electronic health records-based observational study from Northern California, 2015–2020

**DOI:** 10.1186/s13690-023-01095-2

**Published:** 2023-05-06

**Authors:** Robert J. Romanelli, Rivfka Shenoy, Meghan C. Martinez, Satish Mudiganti, Louis T. Mariano, Kyle A. Zanocco, Zachary Wagner, Allison Kirkegaard, Katherine E. Watkins

**Affiliations:** 1 RAND Europe, Cambridge, England; 2 University of California, Los Angeles, CA USA; 3 Sutter Health, Center for Health Services Research—Palo Alto and Walnut Creek, Los Angeles, CA USA; 4 RAND Corporation—Santa Monica, Los Angeles, CA USA

**Keywords:** Opioids, Prescriptions, Surgery, Health care disparities, Electronic health records

## Abstract

**Objectives:**

To examine racial and ethnic disparities in postoperative opioid prescribing.

**Data sources:**

Electronic health records (EHR) data across 24 hospitals from a healthcare delivery system in Northern California from January 1, 2015 to February 2, 2020 (study period).

**Study design:**

Cross-sectional, secondary data analyses were conducted to examine differences by race and ethnicity in opioid prescribing, measured as morphine milligram equivalents (MME), among patients who underwent select, but commonly performed, surgical procedures. Linear regression models included adjustment for factors that would likely influence prescribing decisions and race and ethnicity-specific propensity weights. Opioid prescribing, overall and by race and ethnicity, was also compared to postoperative opioid guidelines.

**Data extraction:**

Data were extracted from the EHR on adult patients undergoing a procedure during the study period, discharged to home with an opioid prescription.

**Principal findings:**

Among 61,564 patients, on adjusted regression analysis, non-Hispanic Black (NHB) patients received prescriptions with higher mean MME than non-Hispanic white (NHW) patients (+ 6.4% [95% confidence interval: 4.4%, 8.3%]), whereas Hispanic and non-Hispanic Asian patients received lower mean MME (-4.2% [-5.1%, -3.2%] and − 3.6% [-4.8%, -2.3%], respectively). Nevertheless, 72.8% of all patients received prescriptions above guidelines, ranging from 71.0 to 80.3% by race and ethnicity. Disparities in prescribing were eliminated among Hispanic and NHB patients versus NHW patients when prescriptions were written within guideline recommendations.

**Conclusions:**

Racial and ethnic disparities in opioid prescribing exist in the postoperative setting, yet all groups received prescriptions above guideline recommendations. Policies encouraging guideline-based prescribing may reduce disparities and overall excess prescribing.

**Supplementary Information:**

The online version contains supplementary material available at 10.1186/s13690-023-01095-2.

## Introduction

In the United States (U.S.), prescription-based opioids have been a major contributor to the opioid epidemic as they carry a risk of dependence and addiction[1, 2] and can lead to use of illicit opioids such as heroin or fentanyl.[3, 4] Long-term opioid use is also associated with gastrointestinal issues, fractures, immunosuppression, and hormonal dysregulation.[5].

In the postoperative setting, opioids are of particular concern. Reports indicate that over half of opioids prescribed for postoperative pain management at discharge go unused,[6, 7] creating a reservoir of pills for potential misuse, abuse, and diversion.[8] In recent years, several institutions have developed postoperative opioid prescribing guidelines for common surgical procedures to reduce excess prescribing and to mitigate risks associated with these medications.[9–14].

Historically, the opioid epidemic has been framed as a public health issue impacting white communities.[15–18] This has in part been attributed to misconceptions among clinicians about pain tolerance and implicit biases about illicit drug use, resulting in lower opioid prescribing to communities of color.[19–23] Numerous studies have reported that non-Hispanic Black (NHB), Asian (NHA), and Hispanic individuals are less likely to receive prescription-based opioids than their non-Hispanic white (NHW) counterparts, or that they are more likely to receive opioid prescriptions in lower pill quantities or of lower potency in ambulatory and emergency department settings, even for conditions that can be definitively diagnosed and are objectively painful (e.g., bone fractures).[24–28] Whereas some have argued that such systematic discrimination may have inadvertently shielded certain groups from the downstream effects of overprescribing, data from the Kaiser Family Foundation suggest that opioid-related deaths have been increasing in communities of color for over a decade.[17].

Prior studies on disparities in postoperative opioid prescribing have focused primarily on in-hospital opioid administration or long-term opioid use.[29, 30] Fewer studies in this setting have examined discharge opioid prescribing for adults, especially across different surgical subspecialties or service lines.[25, 31–33] In this study, we utilized electronic health records (EHR) data from a community-based, multi-hospital healthcare delivery system in Northern California to test the hypothesis that racial and ethnic minority groups receive opioid prescriptions at postoperative discharge with lower morphine milligram equivalents (MME) than NHWs. We further conducted exploratory analyses to examine opioid prescribing by race and ethnicity relative to Mayo Clinic guideline recommendations[11–13] and to identify the source of potential differences in prescribing by race and ethnicity, including temporal trends and whether prescriptions were written within or above clinical guideline recommendations, to help inform future public health policy.

## Methods

### Study setting and design

This study was conducted at Sutter Health, a large, integrated and community-based healthcare delivery system in Northern California (https://www.sutterhealth.org/). Sutter has 24 acute-care hospitals and > 100 ambulatory-care clinics across 22 state counties, composed of both urban and rural communities. The Sutter patient population is diverse in terms of race and ethnicity and is representative of the underlying geographic area.

This study was conducted as a retrospective, observational analysis using data from the Sutter Health EHR (Epic; https://www.epic.com/) between January 1, 2015 and February 2, 2020 (study period). The RAND and Sutter Health Institutional Review Boards approved this study.

### Surgical procedure selection

Our analysis included procedures within service lines that are broadly performed across Sutter hospitals: General Surgery, Orthopedic Surgery, and Obstetrics and Gynecology (Ob/Gyn). We mapped procedures to opioid prescribing guidelines developed by the Mayo Clinic for postoperative, at-home pain management.[11–13] These guidelines recommend 5-mg oxycodone pill quantities, based on consensus of multidisciplinary teams who reviewed patient survey data (***Supplemental Table 1***). We refer to each group of similar procedures associated with a specific guideline as a “guideline-procedure group.”

We selected the most frequent guideline-procedure groups for General Surgery (lumpectomy with or without sentinel lymph node biopsy (lumpectomy +/- SLNB) and laparoscopic appendectomy or cholecystectomy); Orthopedics (knee arthroscopy and total knee arthroplasty [TKA]); and Ob/Gyn (minimally-invasive surgery [MIS] gynecological procedures and Cesarean delivery [C-Section]). These procedures are among the most commonly performed in the United States.[34].

We selected Mayo Clinic guidelines because they most comprehensively covered the surgical procedures of interest compared to other published guidelines. In the case of uncomplicated C-Sections, guidelines followed at the Mayo Clinic are unpublished (personal communication with Professor Elizabeth Habermann, Ph.D., MPH, March 12, 2021); however, they are consistent with guidelines published by the Johns Hopkins University.[10].

### Cohort eligibility criteria

We identified adult patients (≥ 18 years of age) with a surgical procedure within guideline-procedure groups of interest during the study period and an opioid prescription written at hospital discharge. We restricted our analysis to patients with a single surgical procedure during the hospital encounter, a prescription for oral opioid pills at discharge, and those discharged to home. We applied these criteria to exclude more complex cases that may warrant higher opioid quantities than typical cases, or when opioids may be prescribed differently if patients were discharged elsewhere (e.g., nursing facility). Lastly, we excluded patients with prescriptions for > 500 pills (n = 2), as we expected these to be erroneous and would be rejected by dispensing pharmacies.

### Outcome and explanatory variables

Outcomes were total MME[35] and prescriptions within or above guideline recommendations. The main explanatory variable was race and ethnicity. Sutter Health has collected self-reported race and ethnicity data since 2010, based on U.S. Census groupings.[36] For the purposes of this study, we operationalized race and ethnicity as: Hispanic, any race; NHW; NHB; NHA; and non-Hispanic other (NHO). The latter group included patients who self-identified as Native Hawaiian/Pacific Islander, Native American or Alaskan Native, mixed/more than one race, or self-reported as “other.” Individuals whose race/ethnicity were not documented in the EHR were excluded.

### Covariates

We extracted EHR data on patient demographics, clinical characteristics, and encounter characteristics that might influence postoperative prescribing decisions, as well as opioid-prescriber characteristics. Patient demographics included age, sex, smoking status, and insurance payer. We further classified patients as existing primary-care patients within the health system (y/n). Patient clinical characteristics included body mass index (BMI; kg/m^2^), diabetes diagnosis (y/n), any chronic pain diagnosis (y/n), use of an opioid within 12 months prior hospital admission (y/n), concurrent benzodiazepine prescription (y/n), postoperative use/administration of an opioid in the 24 h before hospital discharge (y/n), and American Society of Anesthesiology (ASA) physical status[37] as documented at the time of surgery. We also attempted to identify mood disorders (e.g., major depressive disorder, bipolar disorder) and substance use disorders in the 12 months prior; however, no patients in our cohort had these documented on their problem list at the time of surgery.

Encounter characteristics included procedure name and date; procedure class (elective, emergent, or urgent/trauma); procedure duration; hospital length of stay; and discharge time of day. Opioid-prescriber characteristics included provider type (surgical, hospitalist, primary-care physician, other physician specialty, or advanced-care practitioner), years in practice, and sex. A variable was created to indicate whether the prescribing provider was the same as the surgeon who performed the procedure (y/n). Another variable was created to indicate whether the sex of the prescriber was concordant with the sex of the patient (y/n), as there is evidence that clinicians may prescribe opioid differently to patients on this basis.[38].

Covariates were classified as warranted or unwarranted in affecting opioid prescribing, based on their expected influence on pain level (or a proxy thereof) or patient safety.[13] Warranted covariates included patient age, gender, BMI, diabetes, current smoking, concurrent benzodiazepine prescription, chronic pain status, prior exposure to opioids, ASA physical status, procedure length, duration of hospital stay, guideline-procedure group, procedure type, and procedure month-year (given temporal trends in prescribing). Unwarranted covariates included patient insurance payer, existing health system patient status, and discharge time, as well as prescriber sex, specialty, years in practice, whether the prescriber was the surgeon who performed the procedure, and whether the prescriber’s sex was concordant with the patient’s sex.

### Statistical analyses

Descriptive statistical analyses were performed on all variables. Continuous variables were summarized as means ± standard deviations (SD) and/or medians ± interquartile ranges (IQR). Categorical variables were summarized as percentages. Missing values for categorical variables were set to “missing/unknown.” For BMI, as a continuous variable, missing values (representing 1% of patient observations) and values < 13 or > 100 kg/m^2^ (representing < 1st and > 99th percentile, respectively) were imputed at the group mean.

Differences in prescribed total MME were estimated using propensity-weighted linear regression for each racial and ethnic minority group compared with the NHW group. Because total MME is not a normally distributed variable (data not shown), it was log transformed to meet modeling assumptions.[39] Model coefficients and 95% confidence intervals (CI) were back-transformed and interpreted as mean proportional differences.[40] Race and ethnicity-specific propensity weights were generated using logistic regression, by modelling the odds that an individual belonged to a minority group versus the NHW group, conditional on warranted covariates (as described above). For analyses by each race and ethnic minority group, NHW individuals received a propensity weight equal to the odds of being in the minority group, and minority group individuals received a propensity weight equal to 1. These weights shape the NHW reference group, so it is as similar as possible to the minority group on the warranted covariates. These methods have been described elsewhere.[41].

The main regression model for the primary outcome employs a technique typically referred to as doubly robust;[42] it features included covariates representing warranted variation in opioid prescribing and race and ethnicity-specific propensity-score weights to ensure comparison of equivalent groups. Models were run pooled and separately by guideline-procedure group for each race and ethnic minority group versus the NHW group. Statistical significance was set at an alpha of 0.05. For clinical interpretability, we calculated an approximate absolute difference in prescribed total MME and 5-mg oxycodone-equivalent pills using the unadjusted mean total MME for the NHW group and, from the statistical models, the mean proportional differences estimated for each racial and ethnic minority group versus the NHW group.

We conducted a series of exploratory analyses pooled and by guideline-procedure group. First, we examined opioid prescribing by race and ethnicity relative to guideline recommendations. Based on predicted values of prescribed 5-mg oxycodone-equivalent pills from the main model, we summarized excess pills prescribed relative to guideline recommendations for each racial and ethnic group; values at or below guidelines were set to 0. Second, we explored differences in opioid prescribing by race and ethnicity when prescriptions were within or above guideline recommendations. To this end, we added a variable for Mayo Clinic guideline concordance (y/n) in the main model as an interaction term with the race and ethnicity variable.

Lastly, we explored additional factors that influence differences in opioid prescribing between racial and ethnic groups by examining trends in prescribing disparities over time and by conducting a series of stepwise models and descriptively examining changes in coefficients for each racial and ethnic minority group compared to the NHW group. *Model 1*: race and ethnicity as the only covariate; *Model 2 (main model)*: race and ethnicity + warranted covariates, and race and ethnicity-specific weights; *Model 3*: race and ethnicity + warranted and unwarranted covariates, and race and ethnicity-specific weights. These three models were repeated with opioid-prescribing provider fixed effects (Models 4–6) to control for differences in the distribution of patients by race and ethnicity within different prescribers.

## Results

### Cohort description

A total of 61,564 surgical patients met study eligibility criteria (***Supplementary Fig. 1***). Patients were on average 41.3 years of age and a majority were female (85.6%) (Table 1). Patients most frequently underwent C-Sect. (43.4%), followed by laparoscopic appendectomy/cholecystectomy (29.2%). Patients were most frequently NHW (46.4%); 27.4% were Hispanic, 12.7% were NHA, 6.2% were NHB, and 7.2% were NHO. Age and gender distributions varied by guideline-procedure group (see ***Supplementary Table 2***).

**Table 1:** **Demographics and Clinical Characteristics by Race/Ethnicity for Patients in the Observational Cohort from Northern California, 2015-2020**

	Non-Hispanic White	Hispanic, Any Race	Non-Hispanic, Asian	Non-Hispanic, Black	Non-Hispanic, Other	All Patients
	N=28,586	N=16,846	N=7,819	N=3,850	N=4,463	N=61,564
**Sex, n (%)**						
Male	4,974 (17.4%)	2,143 (12.7%)	793 (10.1%)	317 (8.2%)	637 (14.3%)	8,864 (14.4%)
Female	23,612 (82.6%)	14,703 (87.3%)	7,026 (89.9%)	3,533 (91.8%)	3,825 (85.7%)	52,699 (85.6%)
Missing	0 (0.0%)	0 (0.0%)	0 (0.0%)	0 (0.0%)	1 (0.0%)	1 (0.0%)
**Mean Age, Years (SD)**	45.4 (16.9)	36.1 (12.8)	41.0 (14.0)	37.9 (14.7)	38.4 (14.0)	41.3 (15.7)
**Mean BMI, kg/m** ^**2**^ **(SD)**	30.4 (6.8)	32.6 (6.9)	27.9 (5.3)	33.9 (8.0)	31.4 (7.2)	31.0 (7.0)
**Current smoker, n (%)**	2,399 (8.4%)	756 (4.5)	214 (2.7%)	443 (11.5%)	318 (7.1%)	4,130 (6.7%)
**ASA Physical Status, n (%)**						
Healthy	3,696 (12.9%)	2,092 (12.4%)	1,297 (16.6%)	235 (6.1%)	586 (13.1%)	7,906 (12.8%)
Mild Systemic Disease	17,236 (60.3%)	10,204 (60.6%)	5,120 (65.5%)	2,152 (55.9%)	2,735 (61.3%)	37,447 (60.8%)
Severe Systemic Disease	6,163 (21.6%)	3,672 (21.8%)	978 (12.5%)	1,264 (32.8%)	913 (20.5%)	12,990 (21.1%)
Incapacitating Disease/Moribund	240 (0.8%)	96 (0.6%)	34 (0.4%)	38 (1.0%)	24 (0.5%)	432 (0.7%)
Missing	1,251 (4.4%)	782 (4.6%)	390 (5.0%)	161 (4.2%)	205 (4.6%)	2,789 (4.5%)
**Diabetes, n (%)**	1,753 (6.1%)	1,287 (7.6%)	660 (8.4%)	359 (9.3%)	347 (7.8%)	4,406 (7.2%)
**Pain Condition, n (%)**	13,553 (47.4%)	4,521 (26.8%)	1,906 (24.4%)	1,210 (31.4%)	1,380 (30.9%)	22,570 (36.7%)
**Procedure Length, n (%)**						
<1 h	20,103 (70.3%)	12,437 (73.8%)	5,805 (74.2%)	2,668 (69.3%)	3,283 (73.6%)	44,296 (72.0%)
1 to 2 h	6,496 (22.7%)	3,348 (19.9%)	1,433 (18.3%)	812 (21.1%)	861 (19.3%)	12,950 (21.0%)
>2 to 3 h	1,088 (3.8%)	466 (2.8%)	215 (2.7%)	153 (4.0%)	126 (2.8%)	2,048 (3.3%)
>3 h	421 (1.5%)	191 (1.1%)	102 (1.3%)	104 (2.7%)	56 (1.3%)	874 (1.4%)
Missing	478 (1.7%)	404 (2.4%)	264 (3.4%)	113 (2.9%)	137 (3.1%)	1,396 (2.3%)
**Median Length of Stay, Days (IQR)**	1.5 (0.3-3.3)	2.4 (0.8-3.6)	3.1 (0.8-4.2)	3.0 (1.0-4.2)	2.4 (0.5-4.0)	2.2 (0.3-3.7)
**Opioid Rx Prior to Admission, n (%)**	5,688 (19.9)	2,698 (16.0)	609 (7.8)	742 (19.3)	631 (14.1)	10,368 (16.8)
**Prior Chronic Opioid Use, n (%)**	1,321 (4.6%)	649 (3.9%)	142 (1.8%)	197 (5.1%)	139 (3.1%)	2,448 (4.0%)
**Opioid Rx Prior to Discharge, n (%)**	26,011 (91.0%)	15,187 (90.2%)	6,676 (85.4%)	3,369 (87.5%)	3,899 (87.4%)	55,142 (89.6%)
**Concurrent benzodiazepine, n (%)**	249 (0.9%)	41 (0.2%)	18 (0.2%)	13 (0.3%)	16 (0.4%)	337 (0.6%)
**Procedure, n (%)**						
Knee Arthroscopy	1,754 (6.1%)	515 (3.1%)	97 (1.2%)	104 (2.7%)	176 (3.9%)	2,646 (4.3%)
Total Knee Arthroplasty	1,358 (4.8%)	190 (1.1%)	115 (1.5%)	70 (1.8%)	80 (1.8%)	1,813 (2.9%)
Lumpectomy +/- SLNB	3,236 (11.3%)	697 (4.1%)	998 (12.8%)	394 (10.2%)	293 (6.6%)	5,618 (9.1%)
Lap. Cholecystectomy/Appendectomy	8,959 (31.3%)	5,299 (31.5%)	1,713 (21.9%)	770 (20.0%)	1,245 (27.9%)	17,986 (29.2%)
MIS Gynecological Procedures	3,505 (12.3%)	1,781 (10.6%)	562 (7.2%)	455 (11.8%)	496 (11.1%)	6,799 (11.0%)
Cesarean Delivery	9,774 (34.2%)	8,364 (49.6%)	4,334 (55.4%)	2,057 (53.4%)	2,173 (48.7%)	26,702 (43.4%)
**Case Type, n (%)**						
Elective	16,882 (59.1%)	8,575 (50.9%)	4,331 (55.4%)	2,027 (52.6%)	2,328 (52.2%)	34,143 (55.5%)
Emergent	2,961 (10.4%)	2,517 (14.9%)	945 (12.1%)	585 (15.2%)	618 (13.8%)	7,626 (12.4%)
Urgent/Trauma	4,817 (16.9%)	3,627 (21.5%)	1,635 (20.9%)	865 (22.5%)	1,032 (23.1%)	11,976 (19.5%)
Missing	3,926 (13.7%)	2,127 (12.6%)	908 (11.6%)	373 (9.7%)	485 (10.9%)	7,819 (12.7%)
**Median Month-Year, Quarter (IQR)**	11.0 (6.0-16.0)	11.0 (6.0-16.0)	11.0 (7.0-16.0)	11.0 (7.0-16.0)	12.0 (7.0-16.0)	11.0 (6.0-16.0)

ASA, American Society for Anesthesiology BMI, body mass index; IQR, interquartile range; Lap, laparoscopic; MIS, minimally invasive surgery; Rx, prescription; SD, standard deviation; SLNB, sentinel lymph node biopsy

Racial and ethnic minority groups tended to be younger than NHW patients, more frequently had diabetes, a documented chronic pain condition, and longer hospital stays (Table 1). NHB patients tended to have a higher BMI than other groups and more frequently had diabetes, more severe morbidity per ASA physical status, procedures of longer duration, and were more frequently current smokers versus other groups.

### Main descriptive outcomes

Pooled mean and median prescribed total MME were 197.9 and 150, respectively (≅ 26.4 and 20.0 5-mg oxycodone-equivalent pills) (Table 2). Patients undergoing TKA had the highest mean and median MME, 578.6 and 450, respectively (≅ 77.1 and 60.0 pills), and patients undergoing lumpectomy +/- SLNB had the lowest MME (156.4 and 150.0, respectively; ≅ 20.9 and 20.0 pills). Within each guideline-procedure group, NHB patients received the highest mean total MME compared to all other racial and ethnic groups and, except for C-section, NHA patients had the lowest. No one race or ethnic group appeared to have opioid prescriptions with outlier MME quantities that could be driving higher mean values (see ***Supplemental Fig. 2***).

**Table 2:** **Descriptive Outcomes by Race/Ethnicity and Guideline-Procedure Group for Patients in the Observational Cohort from Northern California, 2015-2020**

	Non-Hispanic White	Hispanic, Any Race	Non-Hispanic Asian	Non-Hispanic Black	Non-Hispanic Other	All Patients
*Pooled Procedures*	N=28,586	N=16,846	N=7,819	N=3,850	N=4,463	N=61,564
Mean Total MME (SD)	210.0 (174.7)	184.9 (124.6)	176.1 (106.6)	215.6 (179.6)	192.5 (170.2)	197.9 (155.5)
Median Total MME (IQR)	150 (120, 225)	150 (120, 200)	150 (100, 200)	150 (150, 250)	150 (112.5, 225)	150 (120, 225)
Mean 5-mg Oxy Pills (SD)	28.0 (23.3)	24.7 (16.6)	23.5 (14.2)	28.7 (23.9)	25.7 (22.7)	26.4 (20.7)
Median 5-mg Oxy Pills (IQR)	20 (16, 30)	20 (16, 26.7)	20 (13.3, 26.7)	20 (20, 33.3)	20 (15, 30)	20 (16, 30)
***Knee Arthroscopy***	**N = 1,754**	** N = 555**	** N = 97**	** N = 104**	** N = 176**	** N = 2,646**
Mean Total MME (SD)	266.4 (221.5)	263.4 (203.6)	261.7 (202.0)	351.6 (270.7)	256.4 (483.6)	268.4 (246.3)
Median Total MME (IQR)	150 (150, 300)	200 (150, 300)	200 (150, 300)	300 (105, 450)	150 (150, 290)	160 (150, 300)
Mean 5-mg Oxy Pills (SD)	35.5 (29.5)	35.1 (27.1)	34.9 (26.9)	46.9 (36.1)	34.2 (64.5)	35.8 (32.8)
Median 5-mg Oxy Pills (IQR)	20 (20, 40)	26.7 (20, 40)	26.7 (20, 40)	40 (14, 60)	20 (20, 38.7)	21.3 (20, 40)
***Total Knee Arthroplasty***	**N = 1,358**	** N = 190**	** N = 115**	** N = 70**	** N = 80**	** N = 1,813**
Mean Total MME (SD)	597.9 (379.5)	545.4 (367.3)	403.7 (233.8)	604.2 (463.2)	558.5 (460.2)	578.6 (381.0)
Median Total MME (IQR)	450 (300, 800)	450 (300, 675)	375 (247.5, 450)	525 (375, 750)	450 (300, 712.5)	450 (300, 750)
Mean 5-mg Oxy Pills (SD)	79.7 (50.6)	72.7 (49.0)	53.8 (31.2)	80.6 (61.8)	74.5 (61.4)	77.1 (50.8)
Median 5-mg Oxy Pills (IQR)	60 (40, 106.7)	60 (40, 90)	50 (33, 60)	70 (50, 100)	60 (40, 95)	60 (40, 100)
***Lumpectomy +/- SLNB***	**N = 3,236**	** N = 697**	** N = 998**	** N = 394**	** N = 293**	** N =5,618**
Mean Total MME (SD)	156.3 (110.3)	160.8 (97.8)	138.9 (94.7)	194.1 (139.9)	155.3 (104.9)	156.4 (109.0)
Median Total MME (IQR)	150 (100, 200)	150 (100, 200)	100 (100, 150)	150 (100, 225)	150 (100, 200)	150 (100, 200)
Mean 5-mg Oxy Pills (SD)	20.8 (14.7)	21.4 (13.0)	18.5 (12.6)	25.9 (16.7)	20.7 (14.0)	20.9 (14.5)
Median 5-mg Oxy Pills (IQR)	20 (13.3, 26.7)	20 (13.3, 26.7)	13.3 (13.3, 20.8)	20.8 (13.5, 30)	20.8 (13.3, 26.7)	20 (13.3, 26.7)
***Laparoscopic*** ***Appendectomy/Cholecystectomy***	**N = 8,959**	** N = 5,299**	** N = 1,713**	** N = 770**	** N = 1,245**	** N = 17,986**
Mean Total MME (SD)	191.9 (131.5)	175.5 (109.7)	155.3 (93.2)	191.4 (230.5)	176.6 (133.9)	182.5 (129.0)
Median Total MME (IQR)	150 (100, 225)	150 (100, 200)	150 (100, 200)	150 (100, 200)	150 (100, 200)	150 (100, 200)
Mean 5-mg Oxy Pills (SD)	25.6 (17.5)	23.4 (14.6)	20.7 (12.4)	25.5 (30.7)	23.5 (17.9)	24.3 (17.2)
Median 5-mg Oxy Pills (IQR)	20 (13.3, 30)	20 (13.3, 26.7)	20 (13.3, 26.7)	20 (13.3, 26.7)	20 (13.3, 26.7)	20 (13.3, 26.7)
***MIS Gynecological Procedures***	**N = 3,505**	** N = 1,781**	** N = 562**	** N = 455**	** N = 496**	** N = 6,799**
Mean Total MME (SD)	166.2 (115.6)	166.8 (122.2)	154.2 (90.2)	187.0 (132.0)	160.5 (105.4)	166.3 (116.1)
Median Total MME (IQR)	150 (100, 200)	150 (100, 180)	150 (100, 150)	150 (100, 210)	150 (100, 150)	150 (100, 200)
Mean 5-mg Oxy Pills (SD)	22.2 (15.4)	22.2 (16.3)	20.6 (12.0)	24.9 (17.6)	21.4 (14.1)	22.2 (15.5)
Median 5-mg Oxy Pills (IQR)	20 (13.3, 26.7)	20 (13.3, 24)	20 (13.3, 20)	20 (13.3, 28)	20 (13.3, 20)	20 (13.3, 26.7)
***Cesarean Delivery***	**N = 9,774**	** N = 8,364**	** N = 4,334**	** N = 2,057**	** N = 2,173**	** N = 26,702**
Mean Total MME (SD)	196.2 (110.4)	183.7 (102.8)	187.8 (96.9)	214.9 (124.6)	195.4 (113.4)	192.3 (107.7)
Median Total MME (IQR)	150 (150, 225)	150 (135, 200)	150 (150, 225)	165 (150, 250)	150 (150, 225)	150 (150, 225)
Mean 5-mg Oxy Pills (SD)	26.2 (14.7)	24.5 (13.7)	25.0 (12.9)	28.7 (16.6)	26.1 (15.1)	25.6 (14.4)
Median 5-mg Oxy Pills (IQR)	20 (20, 30)	20 (18, 26.7)	20 (20, 30)	22 (20, 33.3)	20 (20, 30)	20 (20, 30)

IQR, interquartile range; MIS, minimally invasive surgery; MME, morphine milligram equivalents, Ob/gyn, obstetrics and gynecology; Oxy, oxycodone; SD, standard deviation; SLNB, sentinel lymph node biopsy

### Main model outcomes

**Fig. 1:**
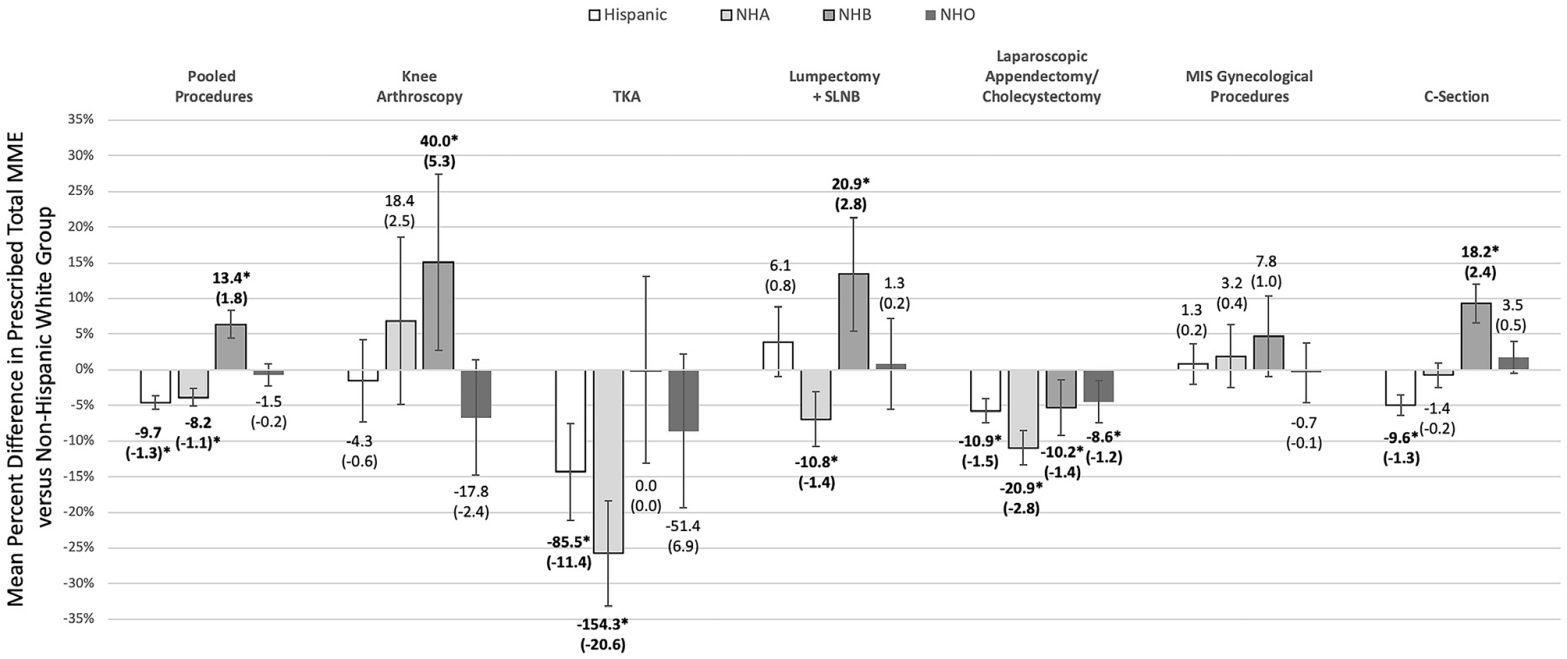
**Opioid Prescribing by Race/Ethnicity and Guideline-Procedure Group. **Mean percent differences in prescribed total morphine milligram equivalents (MME) versus non-Hispanic white group were derived from statistical models, with adjustment and propensity-score weighting for warranted covariates (see Methods section). Error bars represent 95% confidence intervals. Above or below each bar is the calculated, absolute mean difference in total MME and, in parentheses, the mean difference in 5-mg oxycodone equivalent pills, for each racial and ethnic minority group relative to the NHW group based on unadjusted values for NHW patients. *Indicates statistically significant difference, at alpha of 0.05, in bold text. MIS, minimally invasive surgery; NHA, non-Hispanic Asian; NHB, non-Hispanic Black; NHO, non-Hispanic Other; SLNB, sentinel lymph node biopsy; TKA, total knee arthroplasty

After adjusting for warranted covariates, in pooled weighted linear regression analyses, statistically significant differences in opioid prescribing were observed by race and ethnicity. NHB patients received, on average, prescriptions with higher total MME than NHW patients (+ 6.4% [95% CI: 4.4%, 8.3%]), and Hispanic and NHA patients received prescriptions, on average, with lower total MME (-4.2% [-5.1%, -3.2%] and -3.6% [-4.8%, -2.3%], respectively) (Fig. 1). Differences in opioid prescribing by race and ethnicity were also observed within each guideline-procedure group, apart from MIS gynecological procedures. Specifically, mean percent difference in prescribed MME and approximate absolute total MME /5-mg oxycodone-equivalent pills for NHB patients versus NHW patients were higher for knee arthroscopy (+ 15.6% ≅ +45.0 MME or + 6.0 pills), lumpectomy +/- SLNB (+ 12.8% ≅ +20.0 MME or + 2.7 pills), and C-Section (+ 8.8% ≅ +18.1 MME or + 2.4 pills) and were lower for laparoscopic appendectomy/cholecystectomy (-5.6% ≅ -10.5 MME or -1.4 pills). Hispanic patients received lower mean opioid quantities than NHW patients for TKA (-14.5% ≅ -86.9 MME or -11.6 pills), laparoscopic appendectomy/cholecystectomy (-5.0% ≅ -9.7 MME or -1.3 pills), and C-Section (-4.6% ≅ -9.1 MME or -1.2 pills). NHA patients received lower mean opioid quantities relative to NHW patients for TKA (-24.6% ≅ -147.3 MME or -19.6 pills), lumpectomy +/- SLNB (-6.6% ≅ -10.3 MME or -1.4 pills), and laparoscopic appendectomy/cholecystectomy (-10.5% ≅ -20.1 MME or -2.7 pills). Compared to NHW patients, NHO patient received lower mean opioid quantities for laparoscopic appendectomy/cholecystectomy (-4.2% ≅ -8.0 MME or -1.1 pills).

### Exploratory outcomes

#### Guideline-based prescribing

In pooled and unadjusted analyses, 72.8% of all patients received opioid prescriptions at hospital discharge that were above guideline recommendations (Table 3). Statistical models with adjustment for warranted covariates predicted an average excess of 9.0 5-mg oxycodone-equivalent pills prescribed per patient compared to the highest recommended opioid quantity for a given guideline-procedure group. Excess pill quantities were significantly higher among NHB patients (mean of 11.9 pills) and lower among Hispanic patients (mean of 8.5 pills) and NHA patients (mean of 8.7 pills) relative to NHW patients (mean of 9.0 pills). For individual guideline-procedure groups, trends in the relative differences in opioid prescribing by race and ethnicity persisted yet varied in magnitude.

**Table 3:** **Opioid Prescribing Above Guideline Recommendations by Race/Ethnicity for Patients in the Observational Cohort from Northern California, 2015-2020**

	Non-Hispanic White	Hispanic, Any Race	Non-Hispanic Asian	Non-Hispanic Black	Non-Hispanic Other	All Patients
*Pooled Procedures*	N=28,586	N=16,846	N=7,819	N=3,850	N=4,463	N=61,564
Opioid Over-Prescribing, n (%)	20,281 (71.0%)	12,186 (72.3%)	5,978 (76.5%)	3,092 (80.3%)	3,263 (72.9%)	44,880 (72.8%)
Excess 5-mg Oxycodone Pills*						
Predicted Mean (SD)	9.0 (7.7)	8.5 (6.3) ^†^	8.7 (6.1) ^†^	11.9 (7.2) ^†^	8.9 (6.8)	9.0 (7.2)
Predicted Median (IQR)	8.0 (2.9, 13.8)	8.2 (2.8, 13.5)	8.5 (3.9, 13.4)	11.6 (7.0, 16.8)	8.5 (3.5, 13.8)	8.4 (3.3, 13.8)
***Knee Arthroscopy***	**N = 1,754**	** N = 515**	** N = 97**	** N = 104**	** N = 176**	** N = 2,646**
Opioid Over-Prescribing, n (%)	844 (48.1%)	260 (50.1%)	51 (52.6%)	70 (67.3%)	64 (36.4%)	1,289 (48.7%)
Excess 5-mg Oxycodone Pills*						
Predicted Mean (SD)	5.2 (5.8)	4.8 (6.0)	3.9 (5.8)	9.8 (7.3)	4.8 (6.0)	5.2 (6.0)
Predicted Median (IQR)	3.5 (0, 8.3)	2.4 (0, 8.3)	1.1 (0, 5.0) ^†^	9.3 (3.5, 15.0) ^†^	3.0 (0, 7.8)	3.4 (0, 8.4)
***Total Knee Arthroplasty***	**N = 1,358**	** N = 190**	** N = 115**	** N = 70**	** N = 80**	** N = 1,813**
Opioid Over-Prescribing, n (%)	941 (69.3%)	119 (62.6%)	52 (45.2%)	52 (74.3%)	47 (58.8%)	1,211 (66.8%)
Excess 5-mg Oxycodone Pills*						
Predicted Mean (SD)	18.2 (18.2)	17.4 (17.4)	12.1 (14.2) ^†^	22.3 (19.9)	16.1 (17.5)	17.8 (18.0)
Predicted Median (IQR)	12.7 (2.0, 29.7)	13.1 (2.0, 30.2)	6.2(0, 22.6)	19.4 (5.9, 33.1)	9.6 (0.1, 30.2)	12.1 (1.8, 29.7)
***Lumpectomy +/- SLNB***	**N = 3,236**	** N = 697**	** N = 998**	** N = 394**	** N = 293**	** N =5,618**
Opioid Over-Prescribing, n (%)	2,624 (81.1%)	589 (84.1%)	819 (82.1%)	321 (81.5%)	239 (81.6%)	4,592 (81.7%)
Excess 5-mg Oxycodone Pills*						
Predicted Mean (SD)	7.9 (4.2)	7.6 (4.2) ^†^	6.5 (3.6) ^†^	9.4 (4.3) ^†^	7.8 (4.3)	7.7 (4.2)
Predicted Median (IQR)	7.4 (4.4, 10.7)	7.3 (3.9, 10.4)	6.3 (3.6, 9.0)	9.1 (6.0, 12.1)	7.1 (4.4, 10.2)	7.3 (4.3, 10.5)
***Lap. Appendectomy/Cholecystectomy***	**N = 8,959**	** N = 5,299**	** N = 1,713**	** N = 770**	** N = 1,245**	** N = 17,986**
Opioid Over-Prescribing, n (%)	3,829 (42.7%)	1,988 (37.5%)	545 (31.8%)	302 (39.2%)	461 (37.0%)	7,125 (39.6%)
Excess 5-mg Oxycodone Pills*						
Predicted Mean (SD)	3.0 (3.9)	2.2 (3.2) ^†^	1.6 (2.6) ^†^	4.0 (4.6) ^†^	2.4 (3.5) ^†^	2.6 (3.6)
Predicted Median (IQR)	1.2 (0, 5.1)	0 (0, 3.8)	0 (0, 2.6)	0 (0, 3.8)	0 (0, 4.1)	0.6 (0, 4.5)
***MIS Gynecological Procedures***	**N = 3,505**	** N = 1,781**	** N = 562**	** N = 455**	** N = 496**	** N = 6,799**
Opioid Over-Prescribing, n (%)	2,886 (82.3%)	1,522 (85.5%)	458 (81.5%)	395 (86.8%)	420 (84.7%)	5,681 (83.6%)
Excess 5-mg Oxycodone Pills*						
Predicted Mean (SD)	10.0 (5.1)	9.0 (4.85) ^†^	7.3 (4.4) ^†^	12.1 (6.4) ^†^	9.3 (4.8) ^†^	9.6 (5.2)
Predicted Median (IQR)	9.5 (5.7, 13.1)	8.6 (5.1, 12.3)	6.9 (4.1, 10.5)	11.2 (7.3, 15.3)	9.0 (5.4, 12.4)	9.0 (5.5, 12.8)
***Cesarean Delivery***	**N = 9,774**	** N = 8,364**	** N = 4,334**	** N = 2,057**	** N = 2,173**	** N = 26,702**
Opioid Over-Prescribing, n (%)	9,157 (93.7%)	7,708 (92.2%)	4,053 (93.5%)	1,952 (94.9%)	2,032 (93.5%)	24,902 (93.3%)
Excess 5-mg Oxycodone Pills*						
Predicted Mean (SD)	13.9 (4.8)	12.5 (4.4) ^†^	12.2 (4.3) ^†^	15.0 (5.0) ^†^	12.8 (4.7) ^†^	13.2 (4.7)
Predicted Median (IQR)	14.1 (10.0, 17.5)	12.5 (8.8, 15.9)	12.2 (8.8, 15.4)	15.0 (10.8, 18.7)	12.6 (8.8, 16.4)	13.2 (9.4, 16.7)

IQR, interquartile range; Lap, laparoscopic; MIS, minimally invasive surgery; Ob/Gyn, obstetrics and gynecology; SD, standard deviation; SLNB, sentinel lymph node biopsy

*Predicted values derived from main statistical model and set to ‘0’ when predicted value was less than guideline concordant

^†^ Statistically significant different from Non-Hispanic White Group, P<0.05

The interaction between race and ethnicity and guideline-concordant prescribing was statistically significant for NHB and Hispanic patients, such that disparities in opioid prescribing were eliminated when opioid prescriptions were written within the range of 5-mg oxycodone pill quantities recommended by clinical guidelines (***Supplemental Fig. 3***). These interactions were also observed within several guideline-procedure groups (***Supplemental Table 3***).

#### Sources of prescribing variation

In pooled analysis of guideline-procedure groups, patterns in racial and ethnic disparities were largely consistent over the five-year study period, with no observable linear trend (**Supplemental Fig. 4**); however, disparities for NHA patients were not statistically significant in the last observation period between January 2019 and February 2020.

Stepwise models showed that compared to unadjusted estimates, adjusting for warranted covariates alone or in combination with unwarranted covariates could explain much, but not all of the observed differences in opioid prescribing for Hispanic, NHA, and NHO groups versus NHW patients (Model 2 and 3 vs. Model 1; ***Supplemental Fig. 5***). Adjusting for these factors alone did not reduce disparities among NHB patients; however, disparities among NHB patients were mitigated when adjusting for individual providers in combination with warranted covariates alone or in combination with unwarranted covariates (Models 5 and 6 vs. Models 2 and 3; ***Supplemental Fig. 5***). Nevertheless, for all minority patients, except for NHO, disparities in prescribing persisted despite being reduced. Results were similar, although not uniform, within guideline-procedure groups (***Supplemental Table 4***).

## Discussion

In this study, differences in postoperative opioid prescribing at hospital discharge were found among racial and ethnic minority patients relative to NHW patients, overall and for most guideline-procedure groups. Findings among Hispanic and NHA patients are consistent with our hypothesis and a body of literature[21, 25, 31, 43] showing that racial and ethnic minority groups, on average, tend to receive lower opioid prescription quantities than NHW patients overall and for several guideline-procedure groups. Unexpectedly and contrary to our hypothesis, we observed heterogeneity in the direction of disparities for NHB patients. Whereas they received opioid quantities that were, on average, higher than NHW patients overall and for knee arthroscopy, lumpectomy +/- SLNB, and C-sections, they received lower opioid quantities for laparoscopic appendectomy/cholecystectomy. Disparities in prescribing across race and ethnic groups largely persisted over the five-year study period, despite many changes to the culture of opioid prescribing during this time.

To our knowledge, this is the first work that has demonstrated differences in postoperative opioid prescribing within and across service lines by race and ethnicity. While most prior literature has shown lower opioid prescribing quantities among NHB patients relative to NHW patients,[21, 25, 27, 43] our study is consistent with at least one other study by Herb et al. in the postoperative setting.[31] In their study, which focused on postoperative opioid prescribing for general surgeries conducted in late 2018 at a large academic medical center in North Carolina, the authors found that prior to the implementation of standardized opioid prescribing schedules, Black patients received prescriptions that were on average + 18.5 MME higher than white patients. That their study was conducted in a different setting than ours (academic vs. community-based institution) and in a distinct geographic region, supports the need for further investigation into postoperative opioid prescribing for NHB patients.

The opposing direction of observed differences in opioid prescribing among individuals of different racial and ethnic groups (or same racial and ethnic groups receiving different procedures) is a reminder that the mechanisms behind disparities is complex and must be considered within the context of patient, physician, hospital, and other systemic factors.[44, 45] For example, patients may ask for non-opioid alternatives after surgery depending on their receipt of anticipatory guidance, health literacy, or trust in their provider.[46] Implicit or explicit biases may also lead clinicians to prescribe differently to specific groups, related to perceptions about pain tolerance and likelihood for misuse/abuse of prescribed opioids. Bringing attention to such disparities in postoperative opioid prescribing is the first step to making changes.

Differences between racial and ethnic groups in terms of demographic, clinical, and encounter-specific factors explained a large proportion of the disparities in opioid prescribing for Hispanic and NHA patients, and completely for NHO patients, relative to NHW patients. Notably, however, differences in prescribing among NHB patients was most impacted by adjusting for individual opioid-prescribing providers (i.e., provider fixed effects), suggesting that these disparities are in part driven by NHB patients seeing providers with a greater propensity for higher opioid prescribing compared to the other groups.

Despite systematic differences in opioid prescribing by race and ethnicity, excess prescribing was observed across all groups, with approximately 73% of patients receiving prescriptions above guideline recommendations. Such excess prescribing translates to each patient receiving an average of 9 additional 5-mg oxycodone-equivalent pills per prescription. These data indicate that even when a patient belonging to one racial and ethnic group is prescribed a different opioid quantity than another –all else being equal– most patients receive more pills than they are likely to need for pain control. These findings also underscore the limitations of using specific racial and ethnic groups as a benchmark, such that prescriptions of lower opioid quantities versus NHW patients, for example, should not be interpretated as “appropriate” prescribing.

Importantly, racial and ethnic differences for Hispanic and NHB patients versus NHW patients in opioid prescribing were eliminated when prescriptions were written within guideline recommendations. Similar results were found by Herb et al., where the implementation of a standardized postoperative dosing schedule for general surgeries mitigated observed differences between NHB and NHW patients.[31] In their study and ours, this is likely because guidelines offer a narrower range of pill quantities for all patients. While this is unsurprising, it underscores secondary benefits of implementing guideline recommendations; that is, by reducing variation in care, disparities in care can also be mitigated. Whereas it is unclear why these results did not hold for NHA patients, who tended to receive lower opioid quantities than NHWs regardless of whether the prescription was within or above guideline recommendations, there may be other reasons driving specific opioid prescribing practices relative to NHW patients.

From our study, we cannot know why differences in opioid prescribing persisted for racial and ethnic minority groups after adjusting for various factors. It is especially curious that NHB patients appeared to receive higher opioid quantities for several guideline-procedure groups, which is counter to research from other settings, yet consistent with the aforementioned study by Herb and colleagues.[30] Whereas Herb et al. noted that higher opioid prescribing among NHB patients in their study could be attributed to several clinicians with outlier prescribing practices, our data do not support this explanation in terms of the direction and magnitude of observed disparities (see ***Supplementary Fig. 2***).

This study has several limitations. First, because our study design was retrospective and observational, the potential for unmeasured confounding exists. Specifically, while we were able to adjust for numerous factors that could influence opioid prescribing, we did not have comprehensive information on all patients from their primary-care record. For example, this could explain why we were unable to identify diagnoses of mood disorders in our cohort. Postoperative pain may vary based on unmeasured clinical factors (including measures self-reported pain, which are not recorded in the EHR), warranting a higher number of MME/pills. Thus, it is possible that unmeasured comorbid conditions or self-report pain, which could influence opioid prescribing, were more prevalent among NHB patients compared to NHW patients; thus, explaining the observed higher quantity of opioids prescribed. We also did not have information on prescribing provider race and ethnicity, which has been shown to influence opioid prescribing behavior.[47].

Second, we cannot know from this study if differences in opioid prescribing was clinically important or negatively impacted the clinical course of individual patients (it is possible that residual statistically significant differences are not clinically meaningful).

Third, our study was restricted to patients who received an opioid prescription at discharge. We did not study patients who received non-opioid analgesics or no analgesics after surgery because we could not be certain if an opioid was not prescribed, or it was prescribed prior to hospital admission using a medium other than the Sutter EHR.

Fourth, in exploratory analyses we used postoperative opioid prescribing guidelines developed by one institution, the Mayo Clinic. Where guidelines overlapped with other guidelines in terms of surgical procedures, the recommendations were similar, but they were often more restrictive in terms of the range of recommended pill quantities. Of note, the guidelines used in this study were published during the study period. As such, our comparison to guidelines should not be interpreted as a compliance measure; rather, it provided a benchmark for prescribing practices that was independent of race and ethnicity.

Lastly, data from this study are from a community-based healthcare delivery system in Northern California and included select, albeit commonly performed surgical procedures; thus, we cannot know if findings are generalizable to other geographic regions or to other surgical procedures.

Despite limitations, this study has several important strengths. First, we used data from a large, diverse population that is representative of the underlying geographic area. Second, the healthcare system studied is one of the largest in the U.S. and includes multiple hospitals, spanning numerous state counties across rural and urban communities. Third, the lack of postoperative opioid prescribing guidelines in this healthcare system allowed for an examination of natural variation in prescribing behavior. Lastly, our exploratory analyses provide areas of future study that can serve as targets for future public health policy.

In summary, this work is the first to examine disparities in postoperative opioid prescribing by race and ethnicity across service lines, showing broad heterogeneity in the direction and magnitude of such disparities. Given that the majority of opioid prescriptions written in the postoperative setting were above guideline recommendations and that disparities were attenuated for NHB and Hispanic patients when prescriptions were written within guideline recommendations, future policy should focus on encouraging guideline-based postoperative opioid prescribing, dually reducing disparities and mitigating excess pills available in households and communities that have fueled the opioid epidemic.

## Electronic supplementary material

Below is the link to the electronic supplementary material.

Supplementary Material 1

Supplementary Material 2

Supplementary Material 3

Supplementary Material 4

Supplementary Material 5

Supplementary Material 6

## Data Availability

The datasets analyzed during the current study may be available from the corresponding author on reasonable request.
