# Supplementary figures and images for: Disparities in postoperative opioid prescribing by race and ethnicity: an electronic health records-based observational study from Northern California, 2015–2020
Source: Arch Public Health. 2023 May 6;81:83. doi: 10.1186/s13690-023-01095-2 (PMC10163682; doi:10.1186/s13690-023-01095-2)

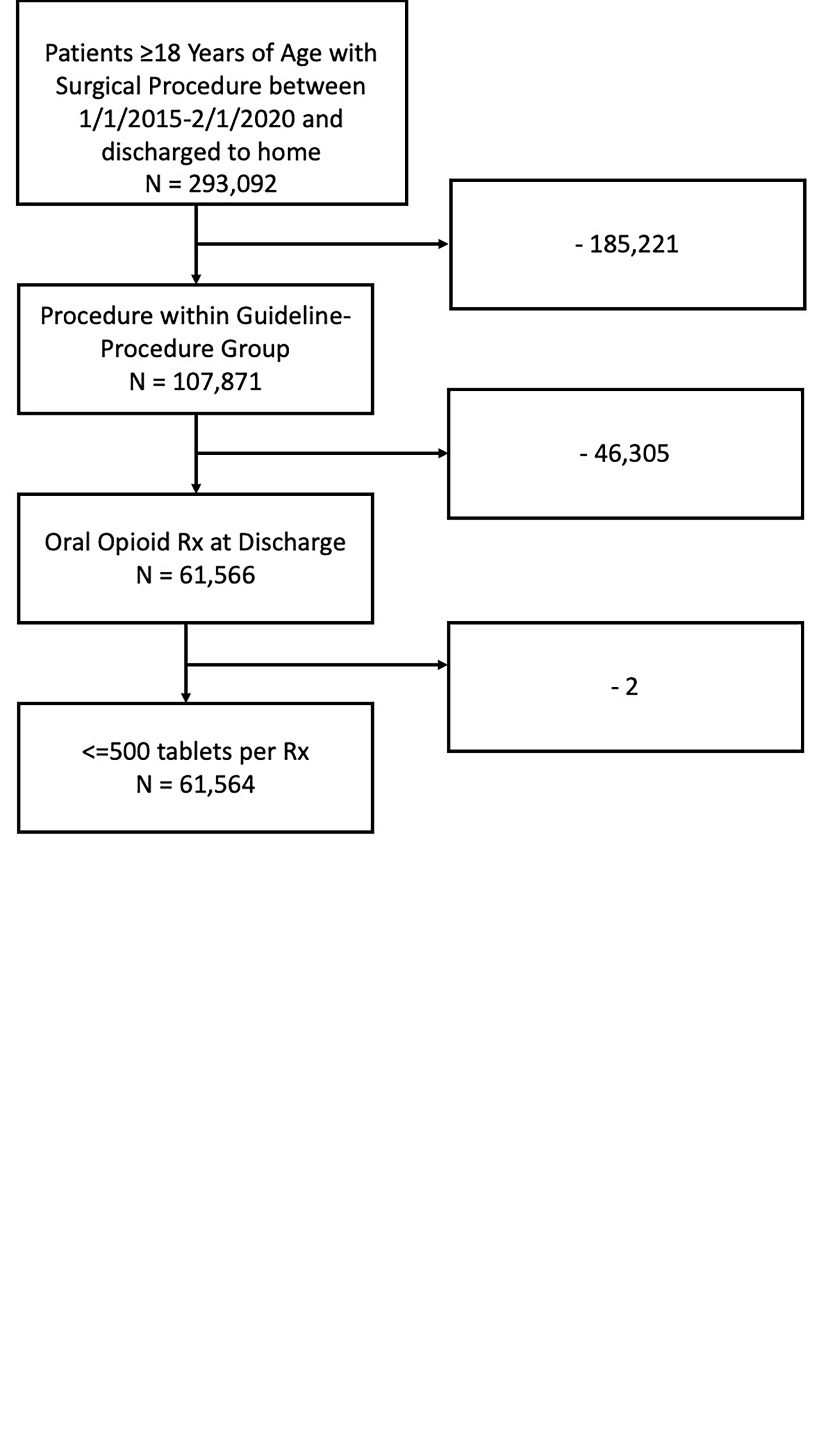

Supplement: Supplementary file 1 — Supplementary Material 1 [file 13690_2023_1095_MOESM1_ESM.jpg]

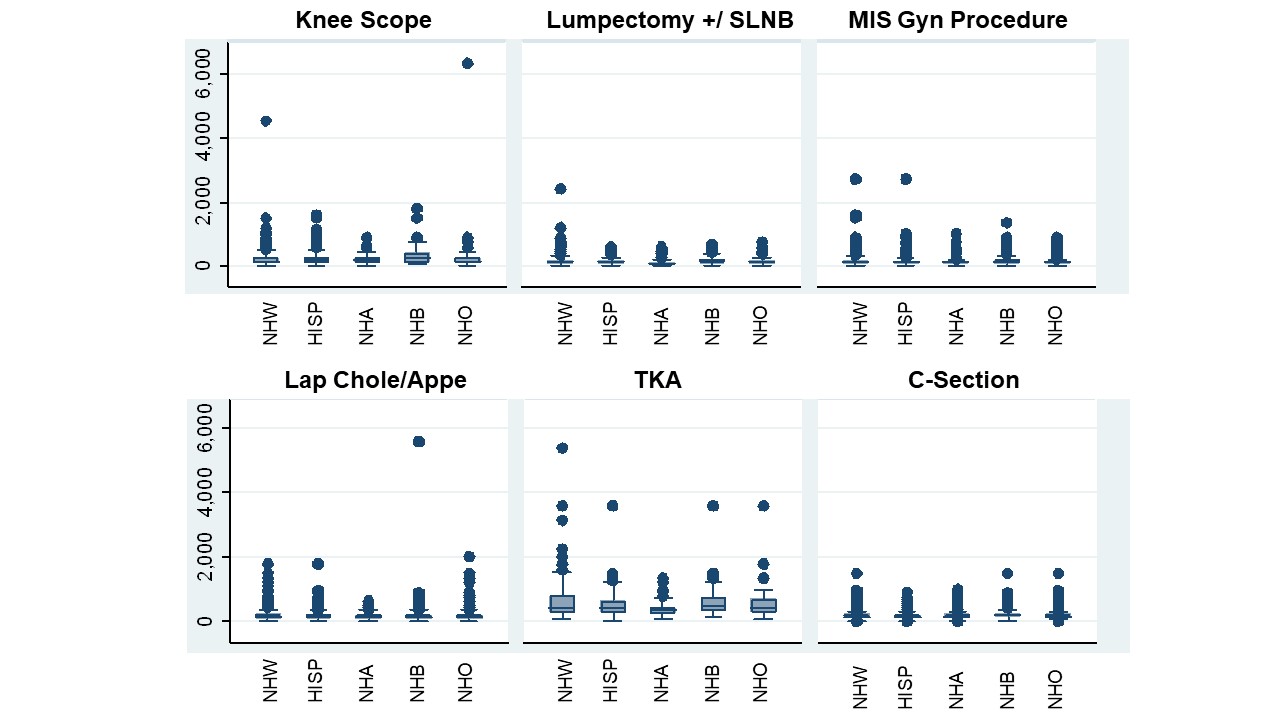

Supplement: Supplementary file 2 — Supplementary Material 2 [file 13690_2023_1095_MOESM2_ESM.jpg]

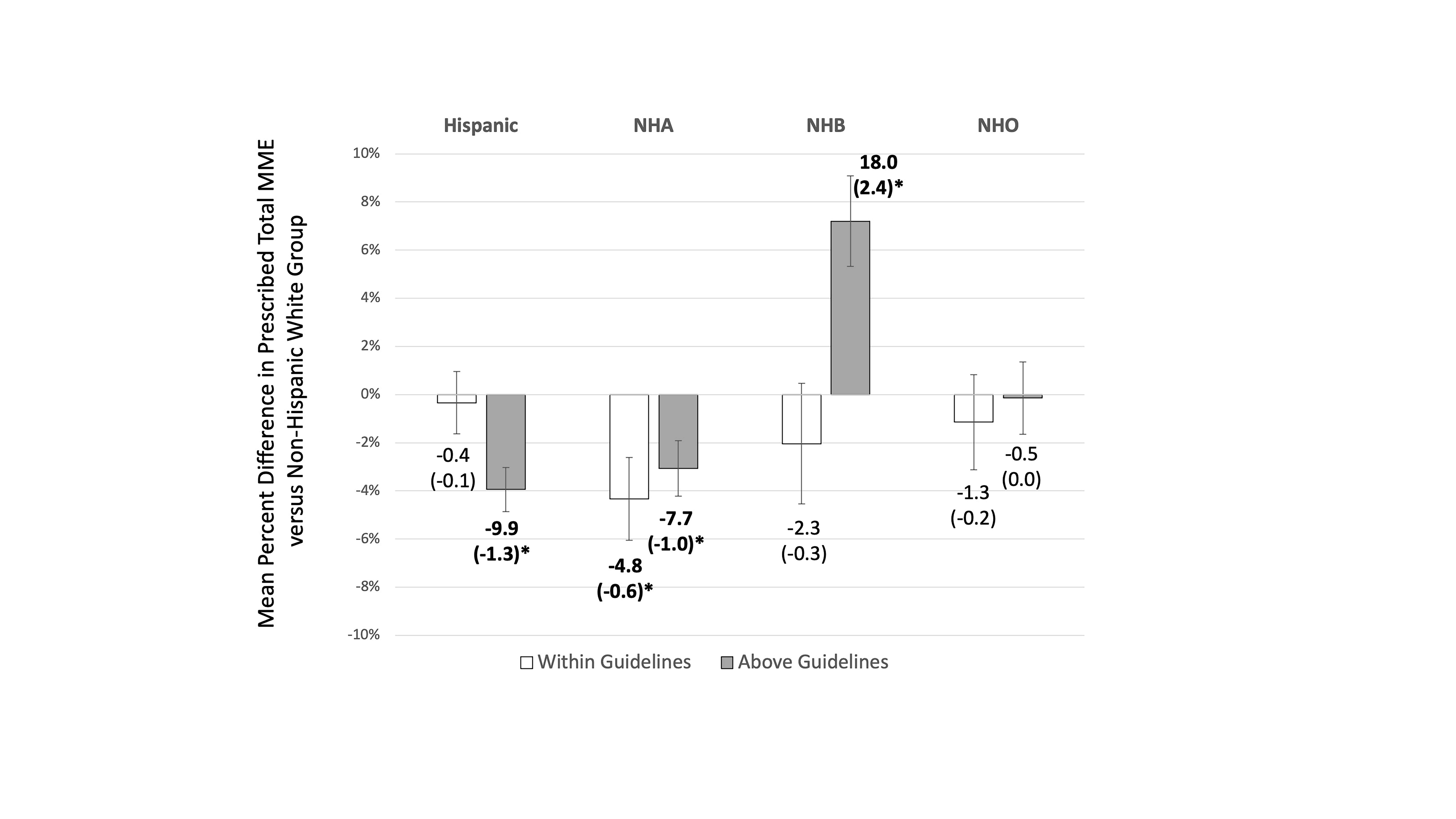

Supplement: Supplementary file 3 — Supplementary Material 3 [file 13690_2023_1095_MOESM3_ESM.jpg]

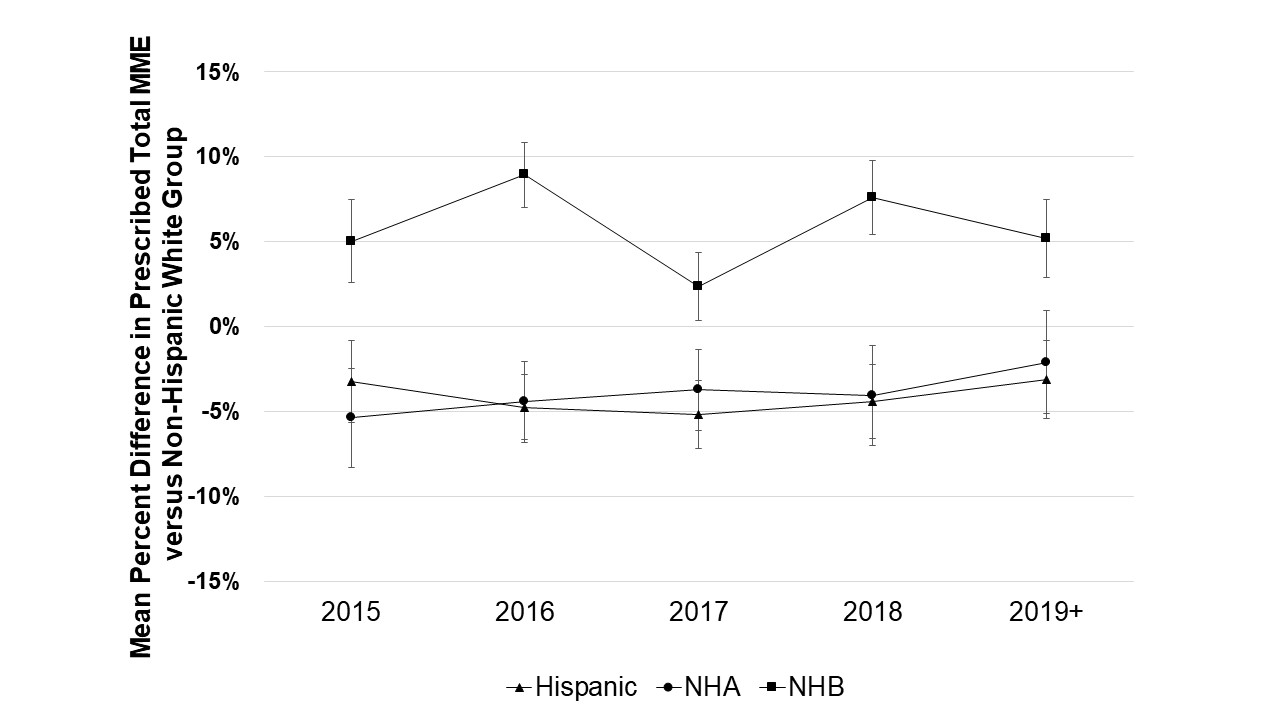

Supplement: Supplementary file 4 — Supplementary Material 4 [file 13690_2023_1095_MOESM4_ESM.jpg]
