# Supplementary material for: Disparities in postoperative opioid prescribing by race and ethnicity: an electronic health records-based observational study from Northern California, 2015–2020
Source: Arch Public Health. 2023 May 6;81:83. doi: 10.1186/s13690-023-01095-2 (PMC10163682; doi:10.1186/s13690-023-01095-2)

**SUPPLEMENTAL TABLES**

**Supplemental Table 1. Mapping of Procedure Names to Guideline-Procedure Group for Mayo Clinic Postoperative Opioid Prescribing Guidelines**

| **Procedure Name** | **Guideline-Procedure  Group** | **5-mg Oxycodone**  **Pill Quantities** | |
| --- | --- | --- | --- |
|  |  | **Standard** | **High** |
| - ARTHROSCOPY KNEE (SPECIFY) W/ ENDOSCOPIC REPAIR - ARTHROSCOPY KNEE W/ MEDIAL PATELLA FEMORAL LIGAMENT RECONSTRUCTION - ARTHROSCOPY KNEE W/ MENISCAL ROOT REPAIR | Knee Arthroscopy [Wyles 2019] | 15 | 25 |
| - ARTHROPLASTY KNEE PATELLOFEMORAL - ARTHROPLASTY KNEE REVISION - ARTHROPLASTY KNEE TOTAL (SPECIFY SIDE) - ARTHROPLASTY KNEE TOTAL BILATERAL - ROBOTIC ARTHROPLASTY KNEE TOTAL | Total Kee Arthroplasty  [Wyles 2019] | 25 | 50 |
| - LAPAROSCOPY APPENDECTOMY - LAPAROSCOPY APPENDECTOMY W/ PARTIAL CECECTOMY - LAPAROSCOPY CHOLECYSTECTOMY BARIATRIC PATIENT - LAPAROSCOPY CHOLECYSTECTOMY COMMON BILE DUCT EXPLORATION - LAPAROSCOPY CHOLECYSTECTOMY W/ OR W/O CHOLANGIOGRAPHY - LAPAROSCOPY SINGLE INCISION CHOLECYSTECTOMY - LESS CHOLECYSTECTOMY - ROBOTIC (SI) LAPAROSCOPIC APPENDECTOMY - ROBOTIC (SI) LAPAROSCOPIC CHOLECYSTECTOMY W/ OR W/O CHOLANGIOGRAMS - ROBOTIC (XI) LAPAROSCOPIC APPENDECTOMY - ROBOTIC (XI) LAPAROSCOPIC CHOLECYSTECTOMY W/ OR W/O CHOLANGIOGRAMS | Laparoscopic Appendectomy/ Cholecystectomy [Thiel 2018] | 8 | 20 |
| - BIOPSY/ EXCISION BREAST MASS / LUMPECTOMY W/ SENTINEL NODE & NEEDLE LOCALIZATION - BIOPSY/ EXCISION BREAST MASS/ LUMPECTOMY W/ SENTINEL NODE & RADAR DETECTION - BIOPSY / EXCISION BREAST - BIOPSY/ EXCISION BREAST / NIPPLE / DUCT - BIOPSY/ EXCISION BREAST MASS/ LUMPECTOMY W/ NEEDLE LOCALIZATION - BIOPSY/ EXCISION BREAST MASS/ LUMPECTOMY W/ RADAR DETECTION - MASTECTOMY PARTIAL / LUMPECTOMY - MASTECTOMY PARTIAL / LUMPECTOMY W/ WIRE LOC W/ SENTINEL NODE BIOPSY - MASTECTOMY PARTIAL/ LUMPECTOMY W/ SENTINEL NODE BIOPSY | Lumpectomy +/- SLNB [Thiel 2018] | 5 | 10 |
| - CLOSURE VESICOUTERINE / VESICOVAGINAL FISTULA LAPAROSCOPIC - LAPAROSCOPY CHROMOTUBATION - LAPAROSCOPY DIAGNOSTIC & HYSTEROSCOPY OPERATIVE - LAPAROSCOPY FULGURATION ENDOMETRIOSIS W/ OR W/O LASER - LAPAROSCOPY HYSTERECTOMY SUPRACERVICAL W/ OR W/O BILATERAL SALPINGO OOPHORECTOMY - LAPAROSCOPY HYSTERECTOMY TOTAL W/ OR W/O BILATERAL SALPINGO OOPHORECTOMY - LAPAROSCOPY HYSTERECTOMY TOTAL W/ OR W/O BILATERAL SALPINGO OOPHORECTOMY & METOIDIOPLASTY - LAPAROSCOPY HYSTERECTOMY TOTAL W/ OR W/O BILATERAL SALPINGO OOPHORECTOMY WITH PELVIC LYMPH NODE DISSECTION - LAPAROSCOPY OVARIAN CYSTECTOMY - LAPAROSCOPY SALPINGO OOPHORECTOMY UNILATERAL / BILATERAL - LAPAROSCOPY SALPINGOTOMY/SALPINGECTOMY REMOVAL ECTOPIC PREGNANCY - LAPAROSCOPY TUBAL LIGATION / SALPINGECTOMY - LAPAROSCOPY W/ DILATATION AND CURETTAGE - LAPAROSCOPY W/ OR W/O SLING - MYOMECTOMY LAPAROSCOPYROBOTIC (SI) LAPAROSCOPIC HYSTERECTOMY SUPRACERVICAL W/ OR W/O BILATERAL SALPINGO OOPHORECTOMY - ROBOTIC (SI) LAPAROSCOPIC HYSTERECTOMY TOTAL W/ OR W/O BILATERAL SALPING OOPHORECTOMY - ROBOTIC (SI) LAPAROSCOPIC HYSTERECTOMY TOTAL W/ OR W/O BILATERAL SALPINGO OOPHORECTOMY & PELVIC LYMPH NODE DISSECTION - ROBOTIC (SI) LAPAROSCOPIC HYSTERECTOMY TOTAL W/ OR W/O BILATERAL SALPINGO OOPHORECTOMY & W/SACROCOLPOPEXY & CYSTOSCOPY - ROBOTIC (XI) LAPAROSCOPIC HYSTERECTOMY SUPRACERVICAL W/ OR W/O BILATERAL SALPINGO OOPHORECTOMY - ROBOTIC (SI) LAPAROSCOPIC MYOMECTOMY - ROBOTIC (SI) LAPAROSCOPIC OVARIAN CYSTECTOMY/OOPHORECTOMY - ROBOTIC (SI) LAPAROSCOPIC SACROCOLPOPEXY & CYSTOSCOPY & POSTERIOR REPAIR & INSERTION SUBURETHRAL SLING - ROBOTIC (XI) LAPAROSCOPIC BILATERAL SALPINGO OOPHORECTOMY - ROBOTIC (XI) LAPAROSCOPIC MYOMECTOMY - ROBOTIC (XI) LAPAROSCOPIC OVARIAN CYSTECTOMY/OOPHORECTOMY - ROBOTIC (XI) LAPAROSCOPIC SACROCOLPOPEXY & CYSTOSCOPY & POSTERIOR REPAIR & INSERTION SUBURETHRAL SLING - ROBOTIC (XI) LAPAROSCOPIC SACROCOLPOPEXY & CYSTOSCOPY - ROBOTIC (XI) LAPAROSCOPIC TRACHELECTOMY | MIS Gynecological Procedure [Glaser, 2020] | n/a | 10 |
| - CESAREAN SECTION - CESAREAN SECTION MULTIPLE BIRTHS – TRIPLETS - CESAREAN SECTION MULTIPLE BIRTHS – TWIN - CESAREAN SECTION REPEAT - CESAREAN SECTION REPEAT W/ TUBAL LIGATION OR BILATERAL SALPINGECTOMY - CESAREAN SECTION W/ TUBAL LIGATION OR BILATERAL SALPINGECTOMY - EMERGENT CSECTION SURGERY ONLY (UPDATE ACTUAL IN LOG) | Cesarean Delivery [personal communication] | n/a | 10 |

MIS, minimally invasive surgery; SLNB, sentinel lymph node biopsy.**Supplemental Table 2. Age and Gender by Guideline-Procedure Groups for Patients in the Observational Cohort from Northern California, 2015-2020**

|  | **Laparoscopic Appendectomy** | **Laparoscopic Cholecystectomy** | **Breast Biopsy/ Lumpectomy** | **Knee  Arthroscopy** | **Total Knee Arthroplasty** | **Caesarian Section** | **MIS Gynecological Procedure** |
| --- | --- | --- | --- | --- | --- | --- | --- |
|  | **N= 6,177** | **N=11,809** | **N= 5,618** | **N=2,646** | **N=1,813** | **N=26,702** | **N=6,799** |
| **Mean Age (SD)** | 40.1 (16.2) | 48.8 (16.9) | 57.7 (15.0) | 51.1 (14.6) | 67.0 (9.2) | 31.7 (5.7) | 43.1 (13.5) |
| **Median Age (IQR)** | 37 (26, 52) | 48 (25, 62) | 59 (47, 69) | 52 (41, 62) | 67 (61, 73) | 32 (28, 36) | 41 (33, 50) |
| **Female, n (%)** | 3,022 (48.9) | 8,307 (70.3) | 5,549 (98.8) * | 1,315 (49.7) | 1,023 (56.4) | 26,700 (99.9) * | 6,783 (99.8) * |

IQR; interquartile range; MIS (Minimally Invasive Surgery); SD, standard deviation.
*A small percentage of patients may have identified as ‘male’ at the time of their procedure.

**Supplemental Table 3 Impact of Prescribing Guideline Concordance on Prescribed Total Morphine Milligram Equivalents by Race/Ethnicity and Guideline-Procedure Group for Patients in the Observational Cohort from Northern California, 2015-2020**

|  | ***Model  Percent differences from NHW (95% CI)*** | | ***Model with Interaction Term: race x guideline Rx Percent differences from NHW (95% CI)*** | | |
| --- | --- | --- | --- | --- | --- |
|  | ***Main Effects*** | ***Main Effects Adj for Compliance*** | **Within-Guideline  Prescribing** | **Above-Guideline Prescribing** | **P-value for Interaction*** |
| **Pooled Procedures** |  |  |  |  |  |
| Hispanic, Any Race | **-4.2% (-5.1%, -3.2%) ^†^** | **-2.7% (-3.4%, -1.9%) ^†^** | -0.2% (-1.5%, 1.2%) | **-3.6% (-4.5%, -2.7%) ^†^** | <0.0001 |
| Non-Hispanic Asian | **-3.6% (-4.8%, -2.3%) ^†^** | **-3.0% (-4.0%, -2.0%) ^†^** | **-4.3% (-6.0%, -2.5%) ^†^** | **-2.7% (-3.9%, -1.6%) ^†^** | 0.159 |
| Non-Hispanic Black | **6.4% (4.3%, 8.3%) ^†^** | **5.4% (3.8%, 7.0%) ^†^** | -2.2% (-4.7%, 0.3%) | **7.2% (5.4%, 9.1%) ^†^** | <0.0001 |
| Non-Hispanic Other | -0.5% (-2.0%, 1.1%) | -0.2% (-1.4%, 1.1%) | -1.0% (-2.9%, 1.0%) | 0.0% (-1.5%, 1.5%) | 0.518 |
| **Knee Arthroscopy** |  |  |  |  |  |
| Hispanic, Any Race | -1.1% (-6.8%, 4.8%) | -1.4% (-5.3%, 2.5%) | 3.4%  (-1.2%, 8.3%) | **-7.8% (-13.6%, -1.6%) ^†^** | 0.010 |
| Non-Hispanic Asian | 7.3% (-3.9%, 19.8%) | -2.0% (-9.2%, 5.6%) | 0.8%  (-7.0%, 9.2%) | -9.3%  (-19.4%, 2.1%) | 0.066 |
| Non-Hispanic Black | **16.9%  (5.3%, 29.8%) ^†^** | 6.2% (-1.2%, 14.1%) | **7.5% (1.4%, 13.9%) ^†^** | 3.0%  (-8.1%, 14.6%) | 0.610 |
| Non-Hispanic Other | -6.5%  (-14.1%, 1.8%) | 2.4% (-3.4%, 8.6%) | 6.5%  (2.0%, 11.1%) | -3.9%  (-14.6%, 8.0%) | 0.158 |
| **Total Knee Arthroplasty** |  |  |  |  |  |
| Hispanic, Any Race | **-14.5%  (-21.6%, -6.9%) ^†^** | **-6.6%  (-12.5%, -0.4 %) ^†^** | -6.5% (-18.6%, 7.4%) | 8.0%  (-13.6, -2.1%) | 0.618 |
| Non-Hispanic Asian | **-24.6% (-31.0%, -17.7%) ^†^** | **-11.7%  (-16.6%, -6.6%) ^†^** | -7.0%  (-14.6%, 1.3%) | **-16.4%  (-22.2%, -10.2%) ^†^** | 0.150 |
| Non-Hispanic Black | 0.7%  (-9.9%, 12.3%) | -1.9%  (-9.5%, 6.4%) | 1.7% (-8.3%, 12.8%) | -5.9%  (-14.3%, 3.2%) | 0.090 |
| Non-Hispanic Other | -9.2%  (-18.6%, 1.3%) | -1.6%  (-8.8%, 6.3%) | 4.9%  (-2.6%, 13.0%) | -1.5%  (-11.9%, 10.1%) | 0.633 |
| **Lumpectomy +/- SLNB** |  |  |  |  |  |
| Hispanic, Any Race | 4.4%  (-0.4%, 9.5%) | 1.7%  (-1.6%, 5.2%) | -1.6% (-7.1%, 4.3%) | 2.7%  (-1.2%, 6.7%) | 0.181 |
| Non-Hispanic Asian | **-6.6%  (-10.3%, -2.6%) ^†^** | **-8.7%  (-11.4%, -5.9%) ^†^** | -3.7%  (-7.9%, 0.8%) | **-9.8%  (-13.0%, -6.5%) ^†^** | 0.016 |
| Non-Hispanic Black | **12.8%  (5.2%, 21.0%) ^†^** | **13.7%  (8.0%, 19.7%) ^†^** | -2.8%  (-9.5%, 4.4%) | **17.1%  (10.1%, 24.6%) ^†^** | 0.002 |
| Non-Hispanic Other | 0.6%  (-5.6%, 7.1%) | -0.1%  (-4.6%, 4.6%) | 2.0%  (-4.1%, 8.6%) | -1.2%  (-6.5%, 4.5%) | 0.177 |
| **Lap Appendectomy/Cholecystectomy** |  |  |  |  |  |
| Hispanic, Any Race | **-5.0%  (-6.8%, -3.3%) ^†^** | **-1.8%  (-3.0%, -0.5%) ^†^** | 0.3%  (-1.3%, 1.9%) | **-4.6%  (-6.4%, -2.7%) ^†^** | <0.0001 |
| Non-Hispanic Asian | **-10.5%  (-12.9%, -8.0%) ^†^** | **-6.4%  (-8.2%, -4.6%) ^†^** | **-6.5%  (-8.8%, -4.2%) ^†^** | **-6.0%  (-8.6%, -3.2%) ^†^** | 0.875 |
| Non-Hispanic Black | **-5.5%  (-9.2%, -1.5%) ^†^** | -1.8%  (-4.5%, 0.9%) | **-3.5%  (-6.7%, -0.1%) ^†^** | -1.1%  (-5.5%, 3.5%) | 0.159 |
| Non-Hispanic Other | **-4.4%  (-7.1%, -1.2%) ^†^** | **-2.2%  (-4.2%, -0.0%) ^†^** | **-2.9%  (-5.5%, -0.2%) ^†^** | -0.9%  (-2.3%, -2.5%) | 0.412 |
| **MIS Gynecological Procedures** |  |  |  |  |  |
| Hispanic, Any Race | 1.4%  (-1.4%, 4.3%) | 0.1%  (-2.1%, 2.4%) | 1.2%  (-3.0%, 5.5%) | -0.1%  (-2.5%, 2.4%) | 0.420 |
| Non-Hispanic Asian | 2.0%  (-2.3%, 6.6%) | 1.1%  (-2.0%, 4.4%) | 1.9%  (-3.3%, 7.4%) | 1.2%  (-2.5%, 5.1%) | 0.860 |
| Non-Hispanic Black | 4.7%  (-0.8%, 10.4%) | 4.0% (-0.3%, 8.5%) | 0.1%  (-7.0%, 7.7%) | **5.0%  (0.1%, 10.2%) ^†^** | 0.117 |
| Non-Hispanic Other | -0.1%  (-4.2%, 4.2%) | -1.8%  (-4.9%, 1.5%) | 4.3% (-0.8%, 9.7%) | -2.7%  (-6.3%, 1.1%) | 0.159 |
| **Cesarean Delivery** |  |  |  |  |  |
| Hispanic, Any Race | **-4.6%  (-6.0%, -3.2%) ^†^** | **-3.9%  (-5.1%, -2.7%) ^†^** | -2.8%  (-5.9%, 0.4%) | **-3.9%  (-5.1%, -2.6%) ^†^** | 0.879 |
| Non-Hispanic Asian | -0.4%  (-2.1%, 1.3%) | -1.0%  (-2.4%, 0.4%) | -0.1%  (-3.9%, 4.2%) | -1.1%  (-2.5%, 0.4%) | 0.846 |
| Non-Hispanic Black | **9.2%  (6.6%, 12.0%) ^†^** | **7.1%  (4.8%, 9.5%) ^†^** | -6.4%  (-12.7%, 0.3%) | **7.9%  (5.5%, 10.4%) ^†^** | 0.007 |
| Non-Hispanic Other | 2.1%  (-0.1%, 4.3%) | 1.0%  (-0.8%, 3.0%) | 1.7%  (-2.6%, 6.2%) | 1.1%  (-0.8%, 3.2%) | 0.689 |

Lap, laparoscopic; MIS, minimally invasive surgery; NHW, non-Hispanic white; Rx, prescribing; SLNB, sentinel lymph node biopsy.
*Stratified analysis with statistically significant interaction terms (P<0.05) are highlighted.
**^†^**Statistically significant from non-Hispanic white group, P<0.05 are in bold text.

**Supplemental Table 4. Stepwise Models* for Percent Differences in Prescribed Total Morphine Milligram Equivalents among Racial/Ethnic Minoritized Groups versus Non-Hispanic White Patients for Patients in the Observational Cohort from Northern California, 2015-2020**

| **Pooled Procedures** | **Model 1** | **Model 2** | **Model 3** | **Model 4** | **Model 5** | **Model 6** |
| --- | --- | --- | --- | --- | --- | --- |
| Hispanic, Any Race | **-6.9% (-8.0%, -5.9%) ^†^** | **-4.2% (-5.1%, -3.2%) ^†^** | **-2.5% (-3.5%, -1.5%) ^†^** | **-2.8% (-3.7%, -1.9%) ^†^** | **-2.4% (-3.3%, -1.6%) ^†^** | **-2.4% (-3.2%, -1.5%) ^†^** |
| Non-Hispanic Asian | **-10.4% (-11.7%, -9.0%) ^†^** | **-3.6% (-4.8%, -2.3%) ^†^** | **-3.5% (-4.8%, -2.3%) ^†^** | **-4.7% (-5.9%, -3.5%) ^†^** | **-2.5% (-3.6%, -1.4%) ^†^** | **-2.5% (-3.6%, -1.4%) ^†^** |
| Non-Hispanic Black | **4.6% (2.4%, 6.8%) ^†^** | **6.4% (4.4%, 8.3%) ^†^** | **6.8% (4.8%, 8.9%) ^†^** | **4.0% (2.2%, 5.8%) ^†^** | **2.6% (0.9%, 4.3%) ^†^** | **2.6% (0.9%, 4.3%) ^†^** |
| Non-Hispanic Other | **-5.3% (-7.1%, -3.4%) ^†^** | -0.5% (-2.0%, 1.1%) | -0.3% (-1.8%, 1.3%) | **-2.3% (-3.7%, -0.8%) ^†^** | -0.3% (-1.5%, 1.0%) | -0.3% (-1.6%, 0.9%) |
| **Knee Arthroscopy** | **Model 1** | **Model 2** | **Model 3** | **Model 4** | **Model 5** | **Model 6** |
| Hispanic, Any Race | 0.6%  (-5.8%, 7.5%) | -1.1%  (-6.8%, 4.8%) | -1.5% (-7.0%, 4.3%) | 1.7%  (-3.7%, 7.4%) | 0.6%  (-4.4%, 6.0%) | 0.6% (-4.4%, 5.9%) |
| Non-Hispanic Asian | 0.4%  (-12.5%, 15.2%) | 7.3%  (-3.9%, 19.8%) | 5.4% (-5.1%, 17.1%) | -1.7%  (-12.0%, 9.8%) | 4.9%  (-3.6%, 14.2%) | 4.5% (-3.8%, 13.5%) |
| Non-Hispanic Black | **33.5%  (16.9%, 52.6%) ^†^** | **16.9%  (5.3%, 29.8%) ^†^** | **18.6% (6.9%, 31.6%) ^†^** | **15.4% (3.2%, 29.0%) ^†^** | **10.5%  (0.4%, 21.6%) ^†^** | **11.4% (1.0%, 23.0%) ^†^** |
| Non-Hispanic Other | -9.2%  (-18.1%, 0.8%) | -6.5%  (-14.1%, 1.8%) | **-9.0% (-15.9%, -1.6%) ^†^** | -5.1%  (-12.7%, 3.1%) | -2.5% (-6.5%, 1.7%) | -2.8% (-9.4%, 4.4%) |
| **Total Knee Arthroplasty** | **Model 1** | **Model 2** | **Model 3** | **Model 4** | **Model 5** | **Model 6** |
| Hispanic, Any Race | **-10.7%  (-18.6%, -2.1%) ^†^** | **-14.5%  (-21.6%, -6.9%) ^†^** | **-9.1%  (-15.9%, -1.8%) ^†^** | -5.0%  (-12.4%, 3.0%) | -4.2% (-10.2%, 2.2%) | -3.9% (-10.1%, 2.7%) |
| Non-Hispanic Asian | **-30.7%  (-38.1%, -22.5%) ^†^** | **-24.6%  (-31.0%, -17.7%) ^†^** | **-13.1%  (-20.3%, -5.3%) ^†^** | **-13.2%  (-21.4%, -4.3%) ^†^** | **-9.8% (-16.1%, -3.1%) ^†^** | **-8.9% (-15.8%, -1.5%) ^†^** |
| Non-Hispanic Black | 1.4%  (-12.0%, 16.9%) | 0.7%  (-9.9%, 12.6%) | 3.3%  (-8.3%, 16.4%) | 8.2% (-5.2%, 23.6%) | 5.9% (-6.1%, 19.3%) | 6.2% (-6.3%, 20.5%) |
| Non-Hispanic Other | -9.2%  (-20.6%, 3.8%) | -9.2%  (-18.6%, 1.3%) | -5.8%  (-14.4%, 3.6%) | -7.8%  (-17.9%, 3.5%) | **-7.9%** **(-14.0%, -1.5%) ^†^** | **-8.3% (-14.0%, -2.2%) ^†^** |
| **Lumpectomy  +/- SLNB** | **Model 1** | **Model 2** | **Model 3** | **Model 4** | **Model 5** | **Model 6** |
| Hispanic, Any Race | 5.1%  (0.0%, 10.5%) | 4.4%  (-0.4%, 9.5%) | 1.1%  (-3.6%, 6.1%) | 0.3%  (-3.3%, 4.0%) | -0.1% (-3.4%, 3.3%) | 0.5% (-3.0%, 4.1%) |
| Non-Hispanic Asian | **-10.0%  (-13.7%, -6.0%) ^†^** | **-6.6%  (-10.3%, -2.6%) ^†^** | -3.4%  (-7.4%, 0.7%) | **-3.8%  (-6.9%, -0.7%) ^†^** | -0.3% (-3.2%, 2.7%) | -0.7% (-3.8%, 2.3%) |
| Non-Hispanic Black | **16.3%  (8.9%, 24.1%) ^†^** | **12.8%  (5.2%, 21.0%) ^†^** | **20.4%  (12.8%, 28.5%) ^†^** | 2.4% (-5.3%, 0.0%) | 0.6% (-4.0%, 5.4%) | 0.5% (-3.9%, 5.2%) |
| Non-Hispanic Other | -0.3%  (-7.3%, 7.2%) | 0.6%  (-5.6%, 7.1%) | -0.8%  (-7.0%, 5.8%) | -2.8%  (-7.8%, 2.5%) | -2.5% (-6.5%, 1.7%) | -2.0% (-6.1%, 2.3%) |
| **Lap Appendectomy/ Cholecystectomy** | **Model 1** | **Model 2** | **Model 3** | **Model 4** | **Model 5** | **Model 6** |
| Hispanic, Any Race | **-6.3%  (-8.1%, -4.5%) ^†^** | **-5.0%  (-6.8%, -3.3%) ^†^** | **-4.5%  (-6.3%, -2.8%) ^†^** | **-2.5%  (-4.0%, -1.0%) ^†^** | **-2.9% (-4.3%, -1.5%) ^†^** | **-3.1% (-4.5%, -1.6%) ^†^** |
| Non-Hispanic Asian | **-17.2%  (-19.7%, -14.7%) ^†^** | **-10.5%  (-12.9%, -8.0%) ^†^** | **-10.0%  (-12.4%, -7.6%) ^†^** | **-6.7%  (-8.9%, -4.4%) ^†^** | **-3.7% (-5.6%, -1.8%) ^†^** | **-3.9% (-5.8%, -2.0%) ^†^** |
| Non-Hispanic Black | -3.8%  (-7.8%, 0.5%) | **-5.5%  (-9.2%, -1.5%) ^†^** | **-5.3%  (-9.0%, -1.5%) ^†^** | **5.8% (2.2%, 9.4%) ^†^** | 0.2%  (-3.1%, 3.6%) | -0.3% (-3.5%, 3.1%) |
| Non-Hispanic Other | **-8.6%  (-11.7%, -5.4%) ^†^** | **-4.4%  (-7.1%, -1.2%) ^†^** | **-3.8%  (-6.6%, -0.9%) ^†^** | **-4.1%  (-6.5%, -1.6%) ^†^** | -1.5% (-3.5%, 0.6%) | -1.8% (-3.8%, 0.2%) |
| **MIS Gynecological Procedures** | **Model 1** | **Model 2** | **Model 3** | **Model 4** | **Model 5** | **Model 6** |
| Hispanic, Any Race | 1.0%  (-2.0%, 4.2%) | 1.4%  (-1.4%, 4.3%) | 1.2%  (-1.6%, 4.2%) | -2.7%  (-5.3%, 0.0%) | -0.3%  (-2.8%, 2.1%) | -0.3% (-2.7%, 2.2%) |
| Non-Hispanic Asian | -4.5%  (-9.0%, 0.2%) | 2.0%  (-2.3%, 6.6%) | 1.9%  (-2.4%, 6.4%) | **-7.9%  (-11.9%, -3.8%) ^†^** | -2.5% (-6.3%, 1.4%) | -2.5% (-6.2%, 1.3%) |
| Non-Hispanic Black | **10.6%  (4.8%, 16.7%) ^†^** | 4.7%  (-0.8%, 10.4%) | **6.2%  (0.7%, 12.0%) ^†^** | 1.8% (-3.2%, 7.1%) | -0.2% (-5.0%, 4.8%) | -0.3% (-5.0%, 4.6%) |
| Non-Hispanic Other | -1.8%  (-6.7%, 3.3%) | -0.1%  (-4.2%, 4.2%) | 0.7%  (-3.2%, 4.8%) | -3.2%  (-7.3%, 1.1%) | -1.3% (-4.8%, 2.3%) | -1.1% (-4.6%, 2.6%) |
| **Cesarean Delivery** | **Model 1** | **Model 2** | **Model 3** | **Model 4** | **Model 5** | **Model 6** |
| Hispanic, Any Race | **-6.4%  (-7.8%, -5.1%) ^†^** | **-4.6% (-6.0%, -3.2%) ^†^** | **-2.8%  (-4.3%, -1.3%) ^†^** | **-3.8% (-5.1%, -2.5%) ^†^** | **-2.8% (-4.3%, -1.4%) ^†^** | **-2.6% (-4.0%, -1.2%) ^†^** |
| Non-Hispanic Asian | **-2.7%  (-4.3%, -1.0%) ^†^** | **-0.4%  (-2.1%, 1.3%) ^†^** | -1.2%  (-3.0%, 0.5%) | **-4.2%  (-5.8%, -2.6%) ^†^** | **-2.7% (-4.0%, -0.9%) ^†^** | **-2.4% (-4.0%, -0.8%) ^†^** |
| Non-Hispanic Black | **8.1%  (5.7%, 10.7%) ^†^** | **9.2%  (6.6%, 12.0%) ^†^** | **10.1%  (7.3%, 13.0%) ^†^** | **4.1% (1.6%, 6.6%) ^†^** | **4.7% (2.2%, 7.1%) ^†^** | **4.8% (2.4%, 7.3%) ^†^** |
| Non-Hispanic Other | -1.0%  (-3.2%, 1.3%) | 2.1%  (-0.1%, 4.3%) | 2.0%  (-0.2%, 4.3%) | -0.6%  (-2.6%, 1.5%) | 1.1%  (-0.9%, 3.1%) | 1.1% (-0.8%, 3.1%) |

Lap, laparoscopic; MIS, minimally invasive surgery; SLNB, sentinel lymph node biopsy.
*Stepwise models -- Model 1: unadjusted; Model 2: adjusted for warranted covariates + race/ethnicity-specific weights; Model 3: adjusted for warranted and unwarranted covariates + race/ethnicity-specific weights; Model 4: Model 1 + adjusted for prescriber fixed effects; Model 5: Model 2 + adjusted for prescriber fixed effects; Model 6: Model 3 + adjusted for prescriber fixed effects. See Methods section for details.
**^†^**Statistically significant from non-Hispanic white group, P<0.05 are in bold text.

**SUPPLEMENTAL FIGURES**

**Supplemental Figure 1. Study eligibility flow diagram.**

**
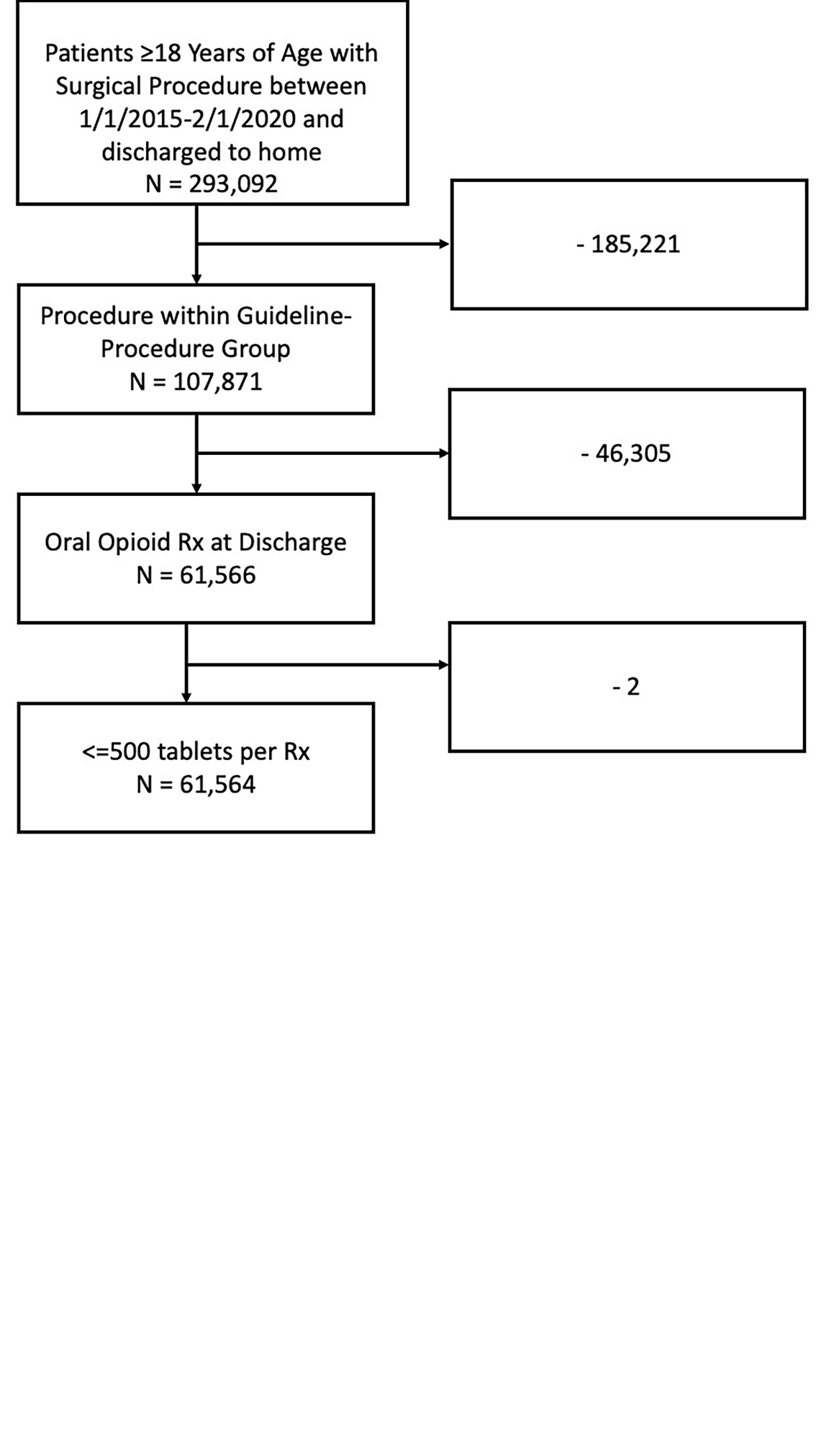
**

**Supplemental Figure 2.** **Distribution of Total Morphine Milligram Equivalents Prescribing by Race/Ethnicity and Guideline-Procedure Group.** MIS, minimally invasive surgery; NHA, non-Hispanic Asian; NHB, non-Hispanic Black; NHO, non-Hispanic Other; NHW, non-Hispanic White; SLNB, sentinel lymph node biopsy; TKA, total knee arthroplasty.


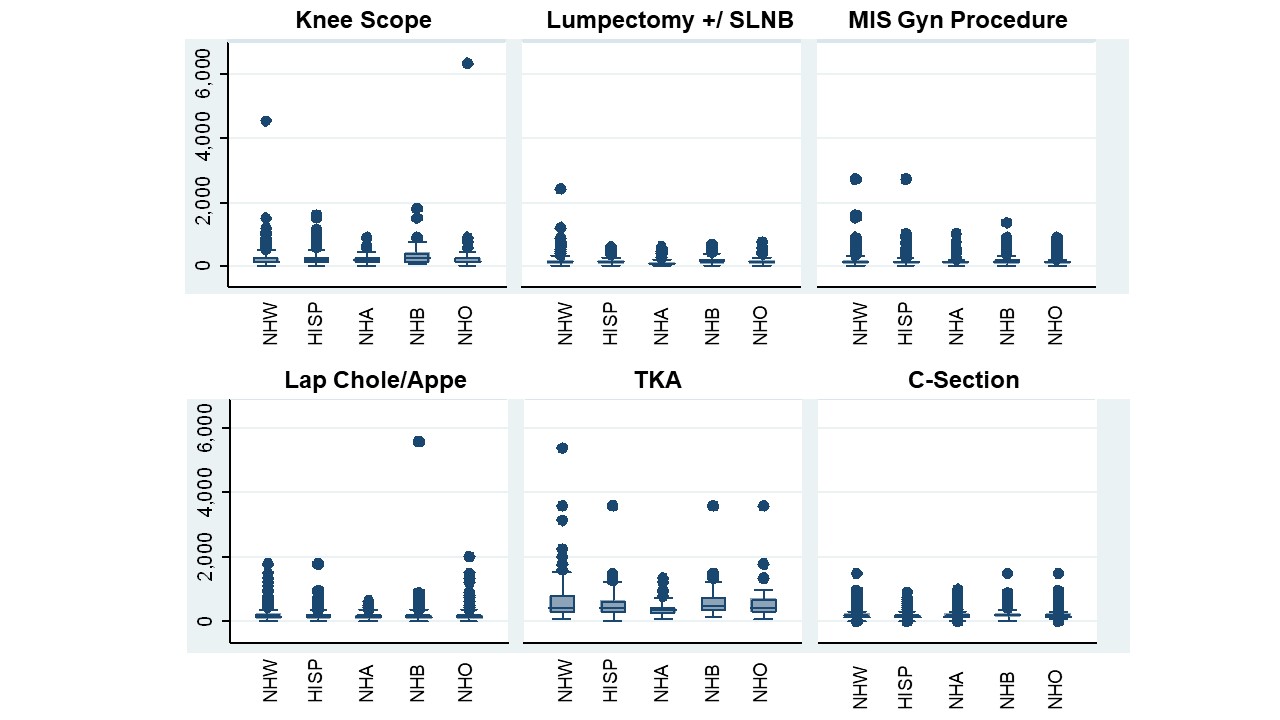


**Supplemental Figure 3. Opioid Prescribing by Race/Ethnicity and Guideline Prescribing.** Mean percent differences in prescribed total morphine milligram equivalents (MME) versus non-Hispanic white group were derived from statistical models, with adjustment and propensity-score weighting for warranted covariates (see Methods section). Error bars represent 95% confidence intervals. Above or below each bar is the calculated, absolute mean difference in total MME and, in parentheses, the mean difference in 5-mg oxycodone equivalent pills, for each racial and ethnic minority group relative to the NHW group based on unadjusted values for NHW patients. *Indicates statistically significant difference, at alpha of 0.05, in bold text. NHA, non-Hispanic Asian; NHB, non-Hispanic Black; NHO, non-Hispanic Other.


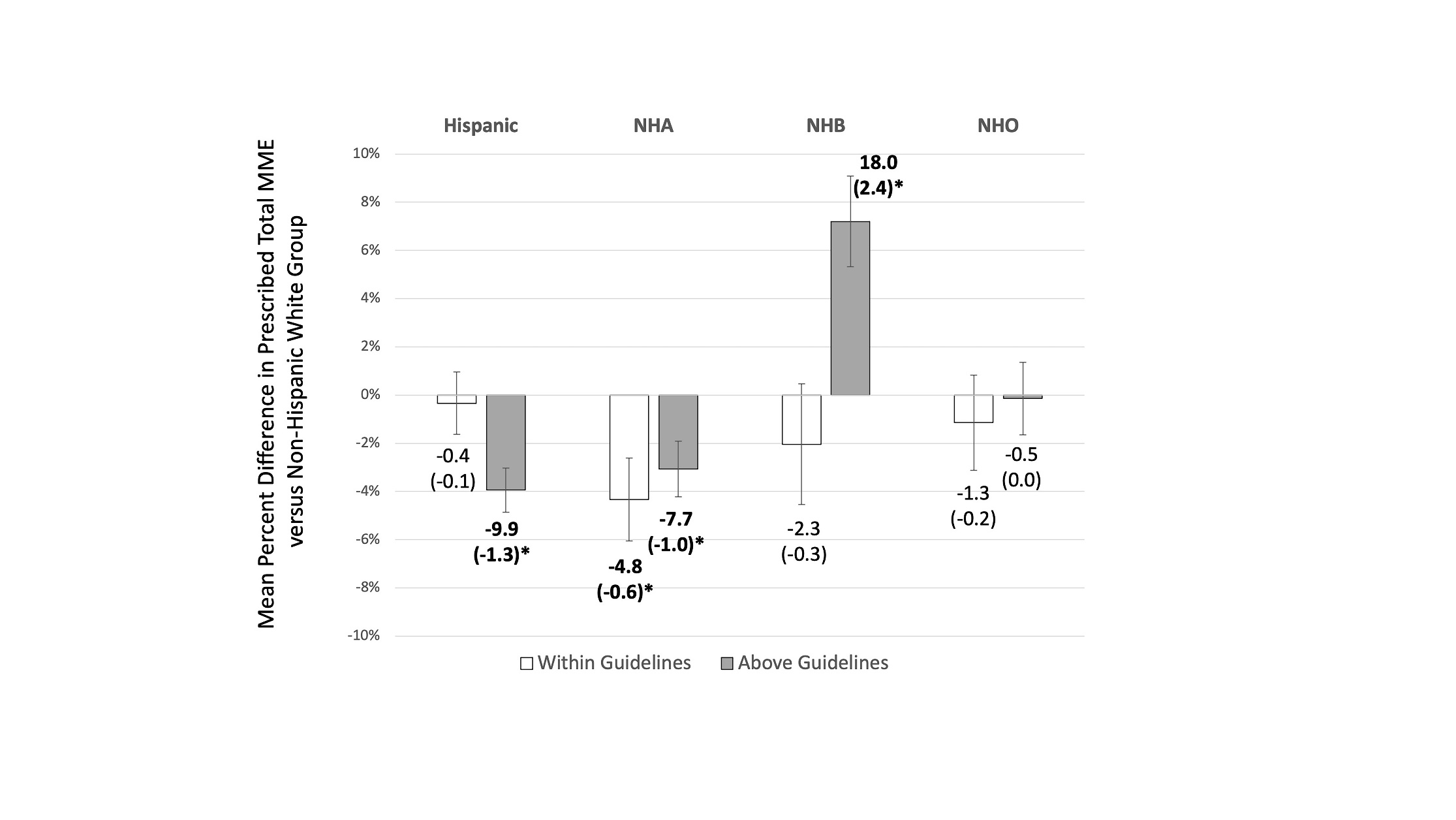


**Supplemental Figure 4. Differences in Total Morphine Milligram Equivalents (MME) Prescribed by Race/Ethnicity and Year.** Differences shown relative to non-Hispanic white group. Error bars represent 95% confidence intervals. NHA, non-Hispanic Asian; NHB, non-Hispanic Black; NHO, non-Hispanic Other.


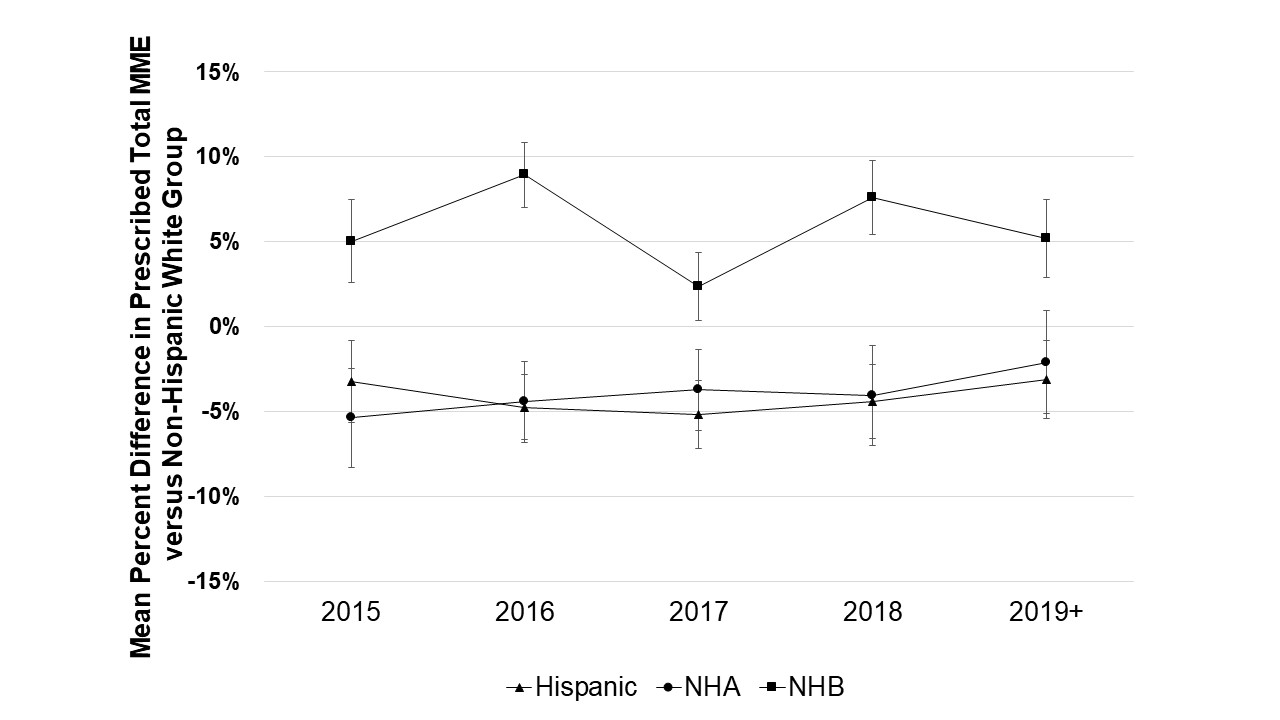


**Supplemental Figure 5.** **Mean percentage difference in prescribed total morphine milligram equivalents (MME) compared to non-Hispanic white group.** Error bars represent 95% confidence intervals. Stepwise models -- Model 1: unadjusted; Model 2: adjusted for warranted covariates + race/ethnicity-specific weights; Model 3: adjusted for warranted and unwarranted covariates + race/ethnicity-specific weights; Model 4: Model 1 + adjusted for prescriber fixed effects; Model 5: Model 2 + adjusted for prescriber fixed effects; Model 6: Model 3 + adjusted for prescriber fixed effects. NHA, non-Hispanic Asian; NHB, non-Hispanic Black; NHO, non-Hispanic Other.


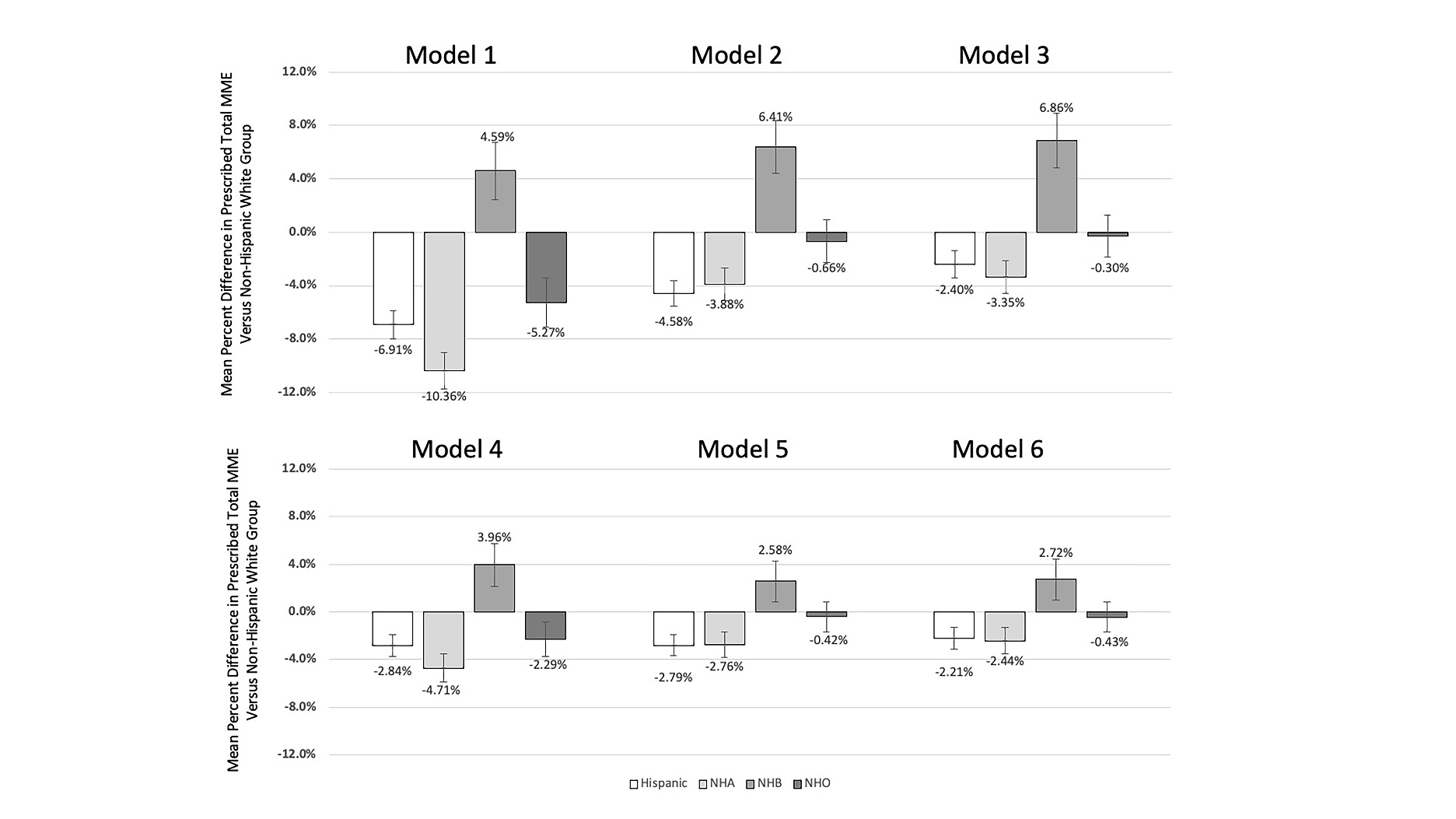

Supplement: Supplementary file 6 — Supplementary Material 6 [file 13690_2023_1095_MOESM6_ESM.docx]
